# Evaluation of stable reference genes for qPCR normalization in circadian studies related to lung inflammation and injury in mouse model

**DOI:** 10.1038/s41598-022-05836-1

**Published:** 2022-02-02

**Authors:** Allan Giri, Isaac Kirubakaran Sundar

**Affiliations:** Division of Pulmonary, Critical Care and Sleep Medicine, Department of Internal Medicine, University of Kansas Medical Center, Kansas City, KS 66160 USA

**Keywords:** Biochemistry, Biological techniques, Molecular biology

## Abstract

Circadian rhythms have a profound effect on lung function and immune-inflammatory response in chronic airway diseases. Thus, understanding the molecular mechanisms of circadian gene expression of core clock-controlled genes (CCGs) may help better understand how it contributes to the physiology and pathology of lung diseases. Ongoing studies have been analyzing gene expression levels of CCGs in mouse lungs using quantitative real-time PCR (qRT-PCR). However, to date, there are no reports on the most stable reference gene in the mouse lung for circadian studies. Herein, we utilized an acute house dust mite (HDM)-sensitization mouse model to evaluate the stability of 10 reference genes commonly used for qRT-PCR normalization using 5 unique algorithms: GeNorm, NormFinder, BestKeeper, RefFinder and Qbase+. *Rn18s* was determined as the most stable reference gene across all samples evaluated, and *Actb*, the least stable reference gene. Furthermore, CircWave analysis showed no diurnal variation in the expression pattern for *Rn18s* but *Actb* showed strong diurnal changes in the lungs of both PBS (control) and HDM groups. We demonstrate systematically how using *Actb* as a housekeeping gene offsets the diurnal expression patterns of the CCGs and leads to statistically significant results which may not be the true reflection of the qRT-PCR analysis.

## Introduction

Entrained by the central nervous system, endogenously generated circadian rhythms in peripheral organs such as the lungs have a well-established role in health and disease^1^. Understanding the molecular mechanisms controlling circadian rhythms have triggered great interest among translational and clinical researchers to understand how altered gene expression of core clock-controlled genes (CCGs: *Clock, Bmal1, Per1/2, Cry1/2, and Rev-erb α/β*) may contribute to the pathophysiology of lung diseases. The circadian clock role and its involvement in lung pathobiology has been previously reviewed^2^. Ongoing studies in the realm of circadian clock disruption in the lung are mostly focused to investigate how these core CCGs alter during the state of a disease. Understanding the normal physiological rhythms of CCGs in the lung will enable us to better define the molecular changes that occur during disease and therefore help devise targeted therapies to selectively modulate circadian genes.

In gene expression analysis, quantitative reverse transcription-polymerase chain reaction (qRT-PCR) is considered the most reliable method for determining the relative mRNA levels. For several decades, qRT-PCR has been used due to its high specificity, sensitivity, accuracy, speed, and higher reproducibility using a minimum amount of RNA^3^. Normalization using a stable reference gene or housekeeping gene (HKG) is an essential step in any qRT-PCR analysis. The inclusion of stable reference genes in the study enables us to account for the sample-to-sample variation and greatly improves the reliability of qRT-PCR assays^4^. Stable reference genes are defined as those that are stably expressed and do not largely vary/differ under different experimental conditions. Unfortunately, there is no single reference gene that is very stable and reliable under all experimental conditions. Hence, it is very important to identify the most stable reference gene for the normalization of qRT-PCR data. This will allow us to interpret our results accurately and avoid misinterpretation of gene expression data.

Prior studies that have determined altered rhythms of CCGs in the lungs have used different reference genes for normalization such as *β-actin* (*Actb*)*, Gapdh, Tbp, Rplp0, Rn18s, 28S*, etc. or geometric mean of 2 or more reference genes (e.g., *Tbp, Hprt1, *and *Rplp0*)^5–12^. Based on our ongoing studies from the mouse model of chronic lung diseases, we found that different agents (such as house dust mite [HDM] allergen or bleomycin exposure) known to induce lung inflammation and injury also affect the transcript levels of both the target (clock genes) as well as the reference genes in a time-dependent manner. We hypothesis that determining the most stable reference gene appropriately for both acute and chronic models will be important before conducting time-dependent change in clock gene expression to determine their role in chronic inflammatory lung diseases. To date, there are no studies that have evaluated the stability of reference genes in the mouse lung for qRT-PCR normalization in circadian studies.

In this study, we utilized an acute HDM-sensitization model to cause allergic airway inflammation and evaluated the stability of 10 most commonly used reference genes using 5 unique algorithms: GeNorm, NormFinder, BestKeeper, RefFinder and Qbase+ (software version 3.0; Biogazelle, Zwijnaarde, Belgium—www.qbaseplus.com)^13–16^. Herein, we found *Rn18s*, as the most stable reference gene to use for acute HDM-sensitization model in the mouse lung tissues across all the samples tested, and *Actb* as the least stable reference gene. We systematically demonstrate how choosing a least stable reference gene can lead to misinterpretation of the qRT-PCR data and suggest that candidate reference genes should always be validated before normalizing with the gene of interest for accurate interpretation of the qRT-PCR data.

## Results

### Expression level of the candidate reference genes

**Figure 1:**
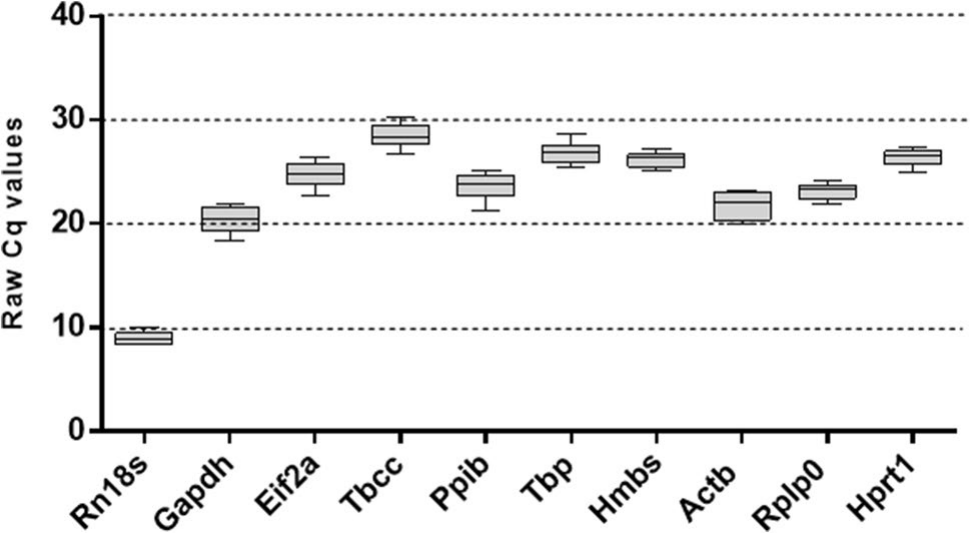
Quantification Cycle (Cq) values of the reference genes across all samples. Box plot for each reference gene representing the interquartile range, median, and the upper and lower range of raw quantification cycle values (Cq) across all samples.

The expression level of the candidate reference genes is represented as the raw quantification cycle (Cq) values. The Cq values of all the 10 reference genes across the samples ranged between 8.3 and 30.2. *Rn18s* showed the lowest Cq value while *Tbcc* showed the highest, indicating that *Rn18s* was the most abundant reference gene in mouse lung samples. The range of the Cq values can help us make preliminary judgments about the expression stability. For instance, *Actb* and *Gapdh*, the most widely used reference genes, had Cq values that ranged from 19.9 to 23.2 and from 18.4 to 21.6 respectively, indicating that the expression of the two references is not consistent throughout all samples. All the raw Cq values are summarized in Fig. 1.

### Primer efficiency

Efficiencies of all the primers used in the study were in the acceptable range of 92% to 109%, and the correlation coefficient (R^2^) with the dilution series were between 0.98 and 0.99 (Table 1). The specific amplification of the target genes was also confirmed by a single peak in the qRT-PCR melt curve analysis.

**Table 1:** Selected mouse housekeeping genes and circadian gene-specific qRT-PCR primer sequences and summary of their amplicon length, efficiency, and correlation coefficient evaluated in this study.

Mouse reference/circadian gene(s)	Gene description	Primer sequences	Amplicon length (bp)	Efficiency (%)	Correlation coefficient (R^2^)
*Rn18s*	18s ribosomal RNA	F: 5′-GTAACCCGTTGAACCCCATT-3′ R: 5′-CCATCCAATCGGTAGTAGCG-3′	151	91.62	0.9941
*Gapdh*	Glyceraldehyde-3-phosphate dehydrogenase	F: 5′-AGGTCGGTGTGAACGGATTTG-3′ R: 5′-TGTAGACCATGTAGTTGAGGTCA-3′	123	99.73	0.9948
*Eif2a*	Eukaryotic translation initiation factor 2a	F: 5′-CACCGCTGTTGACAGTCAGAG-3′ R: 5′-GCAAACAATGTCCCATCCTTACT-3′	142	105.26	0.9986
*Hmbs*	Hydroxymethlbilane synthase	F: 5′-AAGGGCTTTTCTGAGGCACC-3′ R: 5′- AGTGATGAAAGATGGGCAACT -3′	78	103.29	0.9976
*Tbcc*	Tubulin-specific chaperone C	F: 5′-GCGAACAGGAGAGGCAGATAG-3′ R: 5′-GAGTCGTTCAGGAGCTTCCG-3′	208	101.34	0.9888
*Ppib*	Peptidylprolyl isomerase B	F: 5′-GGCTCCGTCGTCTTCCTTTT-3′ R: 5′-ACTCGTCCTACAGATTCATCTCC-3′	122	102.23	0.999
*Tbp*	TATA box binding protein	F: 5′-CTTCCTGCCACAATGTCACAG-3′ R: 5′-CCTTTCTCATGCTTGCTTCTCTG-3′	118	103.34	0.999
*Actb*	Actin, beta	F: 5′-GGCTGTATTCCCCTCCATCG-3′ R: 5′-CCAGTTGGTAACAATGCCATGT-3′	154	90.94	0.9926
*Rplp0*	Ribosomal protein, large, P0	F: 5′-AGATTCGGGATATGCTGTTGGC-3′ R: 5′-TCGGGTCCTAGACCAGTGTTC-3′	109	98.72	0.9994
*Hprt1*	Hypoxanthine phosphoribosyltransferase	F: 5′-CAGTCCCAGCGTCGTGATTA-3′ R: 5′-GGCCTCCCATCTCCTTCATG-3′	167	108.50	0.9954
*Clock*	Clock	F: 5′-GGAGTCTCCAACACCCACAG-3′ R: 5′-GGCACGTGAAAGAAAAGCAC-3′	143	106.10	0.9962
*Arntl1*	Aryl hydrocarbon receptor nuclear translocator-like 1	F: 5′-AAGGGCCACTGTAGTTGCTG-3′ R: 5′-CTGCAGTGAATGCTTTTGGA-3′	147	96.99	0.9893
*Nr1d1*	Nuclear receptor subfamily 1, group D, member1	F: 5′-TGCAGGCTGATTCTTCACACA-3′ R: 5′-AGCCCTCCAGAAGGGTAGGA-3′	89	103.35	0.9949
*Nfil3*	Nuclear factor, interleukin 3, regulated	F: 5′-GAACTCTGCCTTAGCTGAGGT-3′ R: 5′-ATTCCCGTTTTCTCCGACACG-3′	113	106.99	0.9965
*Per2*	Period Circadian Clock 2	F: 5′-CTTGGGGAGAAGTCCACGTA-3′ R: 5′-TACTGGGACTAGCGGCTCC-3′	145	101.89	0.9999
*Cry2*	Cryptochrome 2 (photolyase-like)	F: 5′-TCCCCGGACTACAAACAGAC-3′ R: 5′-GTCTACATCCTCGACCCGTG-3′	135	93.02	0.9903

### Analysis of gene expression stability

**Figure 2:**
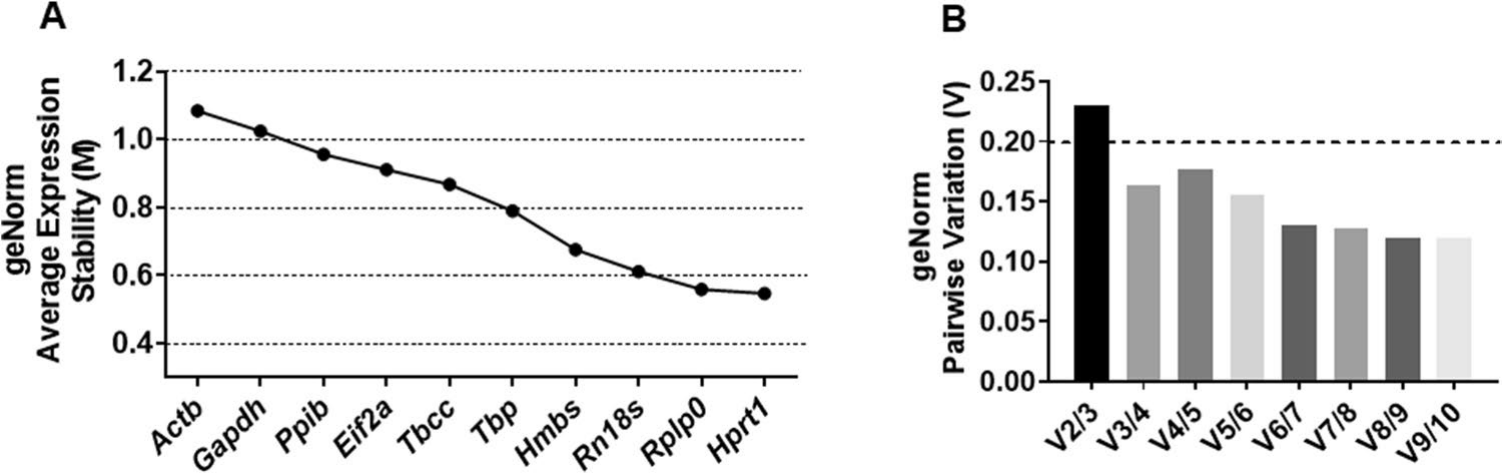
Gene expression stability and optimal number of reference genes required for accurate normalization using GeNorm. (**A**) The geNorm algorithm ranks the reference genes by stepwise exclusion of the least stable reference genes, with the least stable reference gene having the highest M value and vice versa. (**B**) Pairwise variation (Vn/Vn + 1) analysis by geNorm determines the optimal number of genes required for accurate normalization. geNorm V < 0.20 is considered acceptable. The value V3/4 is 0.16, which indicates that the inclusion of the fourth reference gene is not needed for accurate normalization.

**Figure 3:**
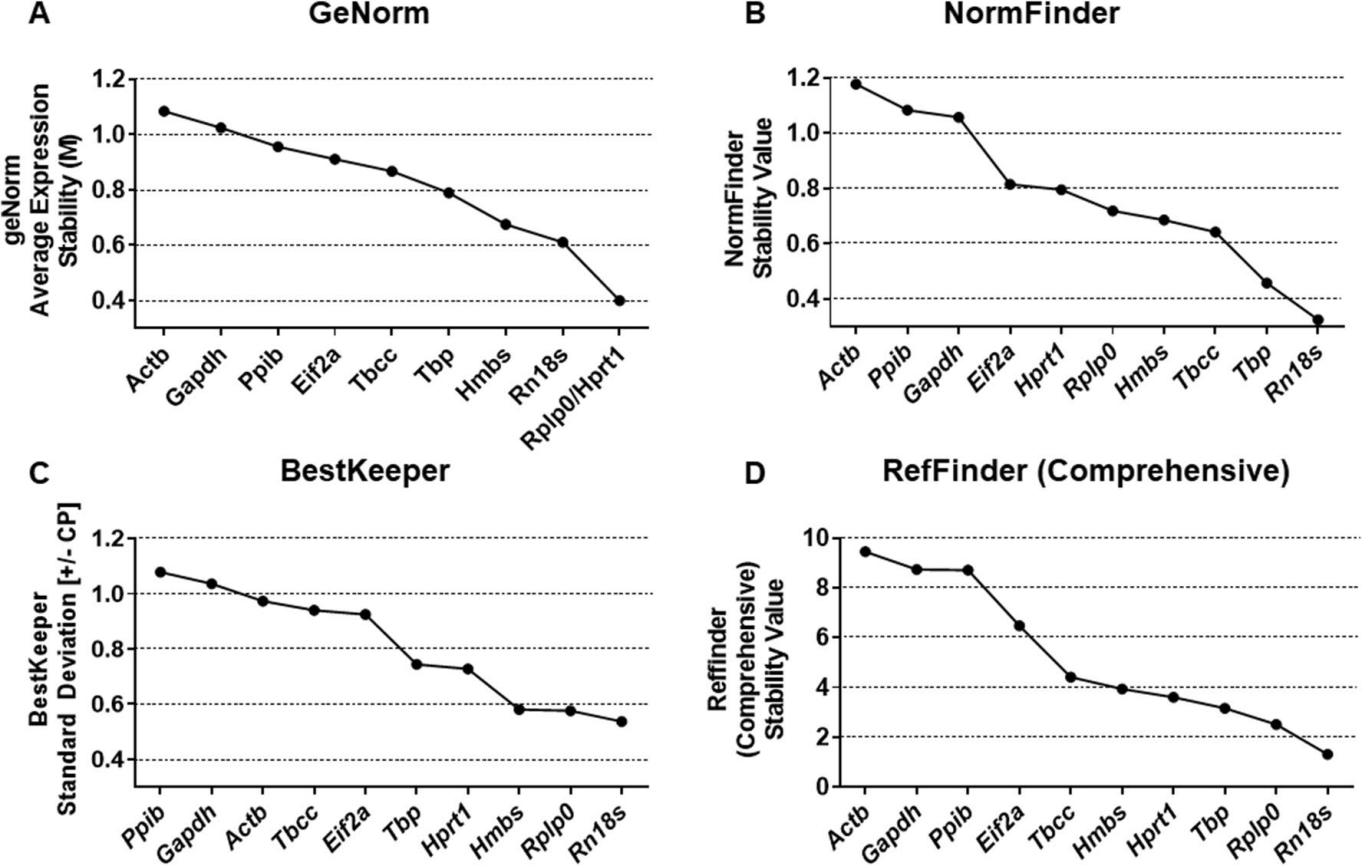
Determination of the most stable reference gene by geNorm (RefFinder), NormFinder, BestKeeper, and RefFinder (Comprehensive). The lower the stability value, the more stable the reference gene according to (**A**) GeNorm and (**B**) NormFinder. RefFinder’s GeNorm algorithm also gives us the best combination of genes that can be used for accurate normalization. (**C**) Reference genes with high standard deviation (SD) and Coefficient of Variation (CV) is considered unstable for normalization according to BestKeeper. Lower the SD value, greater the stability. (**D**) RefFinder provides a final comprehensive ranking by combining the results obtained from GeNorm, NormFinder, BestKeeper and also Delta-Ct (not shown).

The GeNorm algorithm ranks the reference genes by the stepwise exclusion of the least stable reference genes, with the least stable reference gene having the highest M value and vice versa. An M-value less than 0.5 is indicative of a very stable reference gene, while 0.5 < M < 1.0 indicates medium reference target stability. GeNorm analysis using Qbase+ revealed *Hprt1* (0.546) as the most stable reference gene and *Actb* (1.084) as the least stable reference gene for normalization. Expression stability by GeNorm was ranked in the order of least stable to most stable reference genes: *Actb* < *Gapdh* < *Ppib* < *Eif2a* < *Tbcc* < *Tbp* < *Hmbs* < *Rn18s* < *Rplp0* < *Hprt1*. Pairwise variation (Vn/Vn + 1) analysis by GeNorm determines the optimal number of genes required for accurate normalization. GeNorm V < 0.20 is considered acceptable^17^. The value V3/4 is 0.16, which indicates that the inclusion of the fourth reference gene is not needed for accurate normalization (Fig. 2). GeNorm analysis using RefFinder gave us a similar result, except, it also gave us the best combination of two genes (*Rplp0* + *Hprt1*) that can be used for normalization. Here, the stability value of the *Rplp0* + *Hprt1* was 0.400, which is indicative of a strong reference target for normalization (Fig. 3A).

Similar to GeNorm, the lower the stability value obtained by NormFinder, the higher the stability of the candidate reference genes. NormFinder analysis revealed *Rn18s* (0.324) as the most stable reference gene for accurate normalization. *Actb* (1.177) was determined to be the least stable reference gene using NormFinder. Expression stability by NormFinder was ranked as follows: *Actb* < *Ppib* < *Gapdh* < *Eif2a* < *Hprt1* < *Rplp0* < *Hmbs* < *Tbcc* < *Tbp* < *Rn18s* (Fig. 3B).

BestKeeper analyzes the stability based on the standard deviation (SD) and Coefficient of Variation (CV). Reference genes with high SD and CV are considered unstable for normalization. The lower the SD value, the greater the stability. BestKeeper analysis revealed *Rn18s* and *Ppib* with a SD value of 0.537 and 1.078 are the best and least stable reference genes for normalization respectively. Generally, a SD value of more than 1 is considered unacceptable for accurate normalization. Expression stability by BestKeeper was ranked as follows: *Ppib* < *Gapdh* < *Actb* < *Tbcc* < *Eif2a* < *Tbp* < *Hprt1* < *Hmbs* < *Rplp0* < *Rn18s* (Fig. 3C).

The top 4 or 5 reference genes are consistently ranked the highest using all the three algorithms (GeNorm, NormFinder and BestKeeper), except the orders were slightly different (Table 2). For instance, *Rn18s* was ranked as the most stable reference gene by both NormFinder and BestKeeper. According to GeNorm analysis using (Qbase+), *Rn18s* was the third-best reference gene for normalization. Similarly, *Rplp0* was ranked as the second most stable reference gene according to GeNorm (Qbase+) and BestKeeper, but the fifth-best according to NormFinder. RefFinder provides a final comprehensive ranking combining the results obtained from GeNorm, NormFinder, BestKeeper and also Delta-Ct (not evaluated in this study). The comprehensive expression stability determined by RefFinder is as follows: *Actb* < *Gapdh* < *Ppib* < *Eif2a* < *Tbcc* < *Hmbs* < *Hprt1* < *Tbp* < *Rplp0* < *Rn18s* (Fig. 3D).

**Table 2:** Gene expression stability ranking by four different algorithms used in this study.

Group	Rank	GeNorm		Normfinder		Bestkeeper		Reffinder	
		Gene(s)	Stability	Gene(s)	Stability	Stability	SD (± CP)	Gene(s)	Stability
Overall	1	*Rplp0*	0.4	*Rn18s*	0.325	*Rn18s*	0.537	*Rn18s*	1.316
	2	*Hprt1*	0.4	*Tbp*	0.458	*Rplp0*	0.576	*Rplp0*	2.515
	3	*Rn18s*	0.611	*Tbcc*	0.642	*Hmbs*	0.581	*Tbp*	3.162
	4	*Hmbs*	0.675	*Hmbs*	0.686	*Hprt1*	0.728	*Hprt1*	3.6
	5	*Tbp*	0.789	*Rplp0*	0.719	*Tbp*	0.744	*Hmbs*	3.936
	6	*Tbcc*	0.867	*Hprt1*	0.795	*Eif2a*	0.925	*Tbcc*	4.409
	7	*Eif2a*	0.911	*Eif2a*	0.815	*Tbcc*	0.94	*Eif2a*	6.481
	8	*Ppib*	0.957	*Gapdh*	1.057	*Actb*	0.973	*Ppib*	8.712
	9	*Gapdh*	1.024	*Ppib*	1.083	*Gapdh*	1.036	*Gapdh*	8.739
	10	*Actb*	1.084	*Actb*	1.177	*Ppib*	1.078	*Actb*	9.457

### Relative expression of CCGs

**Figure 4:**
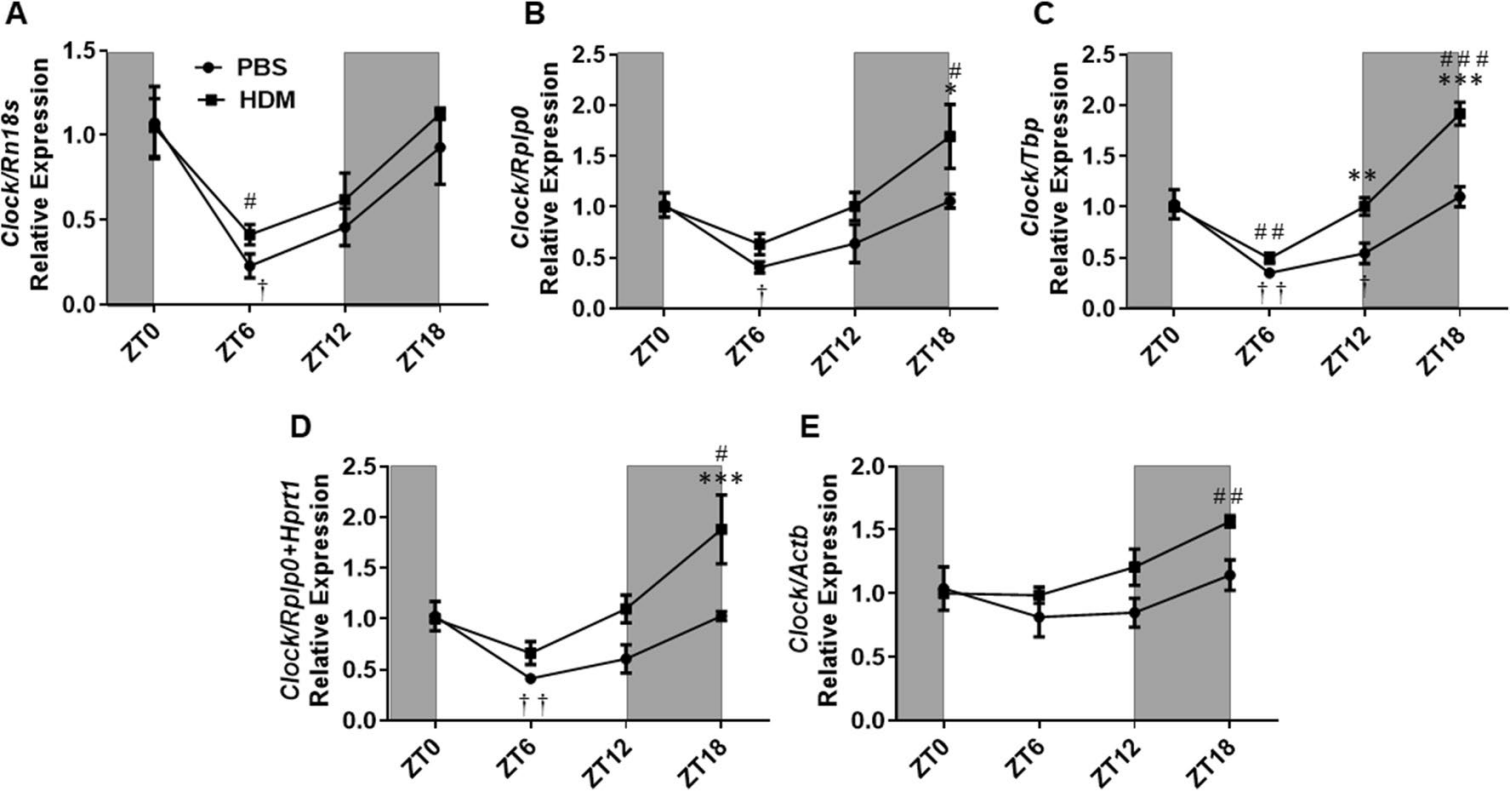
*Clock* gene relative expression in HDM versus PBS exposed mouse lung and data normalized with different reference genes. (**A**) *Rn18s*, (**B**) *Rplp0*, (**C**) *Tbp*, (**D**) *Rplp0 + Hprt1, and* (**E**) *Actb*. Line graphs showing the relative expression of the *Clock* gene in both HDM versus PBS group. White and gray areas in the graph represents the light and dark cycle, respectively. Symbols † and # represent the statistical significance in temporal expression of *Clock* in the PBS and HDM group respectively at the marked time point when compared to the baseline (ZT0). Data are shown as mean ± SEM (n = 4/group; * *P* < 0.05, ** *P* < 0.01, *** *P* < 0.001, compared to respective control; † *P* < 0.05, †† *P* < 0.01, compared to ZT0 PBS; # *P* < 0.05, # # *P* < 0.01, # # # *P* < 0.001, compared to ZT0 HDM).

The top four most stable (*Rn18s, Rplp0*, *Tbp* and *Hprt1*) and the least stable (*Actb*) reference gene determined by RefFinder, and the best combination of gene (*Rplp0* + *Hprt1*) determined by GeNorm was used to normalize the expression patterns of CCGs in experimental groups (PBS and HDM). For *Hprt1* analysis, refer to the supplemental data (Supplementary Figure S1). We found that acute exposure to HDM during the sensitization phase (6 h post-exposure) shows a general trend of increased expression of the *Clock* gene as compared to the PBS control. This increased relative expression is reflected by the difference in amplitude between the PBS and HDM group when normalized with the stable reference genes *Tbp*, *Rplp0,* and *Rplp0* + *Hprt1.* However, amplitude did not change significantly between the HDM and PBS groups when normalized with *Rn18s* (Supplementary Table S1). Statistical significance in the relative expression of *Clock* between the HDM and PBS group was only observed at ZT12 using *Tbp* and *Hprt1* as reference genes, and at ZT18 using *Rplp0, Tbp, Rplp0* + *Hprt1,* and *Hprt1*. No significance was observed using *Rn18s,* the most stable reference gene determined by NormFinder, BestKeeper and RefFinder (Comprehensive) analyses (Fig. 4 and Supplementary Figure S1). Temporal variation analysis of the *Clock* gene revealed a significant decrease in expression at ZT6 versus ZT0 in the PBS group when normalization was performed using top 4 (*Rn18s, Rplp0, Tbp, Hprt1*) and *Rplp0* + *Hprt1* as the reference genes. However, the observed significant decrease in temporal variation was abolished when *Actb* was used for normalization. The only other time points that showed a significant decrease in the gene expression of *Clock* in the PBS group was observed at ZT12 when *Tbp and Hprt1* were used for normalization (Fig. 4 and Supplementary Figure S1). In the HDM group, a significant decrease at ZT6 versus ZT0 was observed when normalization was performed using *Rn18s* and *Tbp*. However, we also found a statistically significant increase in the temporal expression of the *Clock* gene at ZT18 versus ZT0 in the HDM group when normalization was performed using *Rplp0, Tbp, Hprt1, Rplp0* + *Hprt1,* and *Actb* (Fig. 4 and Supplementary Figure S1).

**Figure 5:**
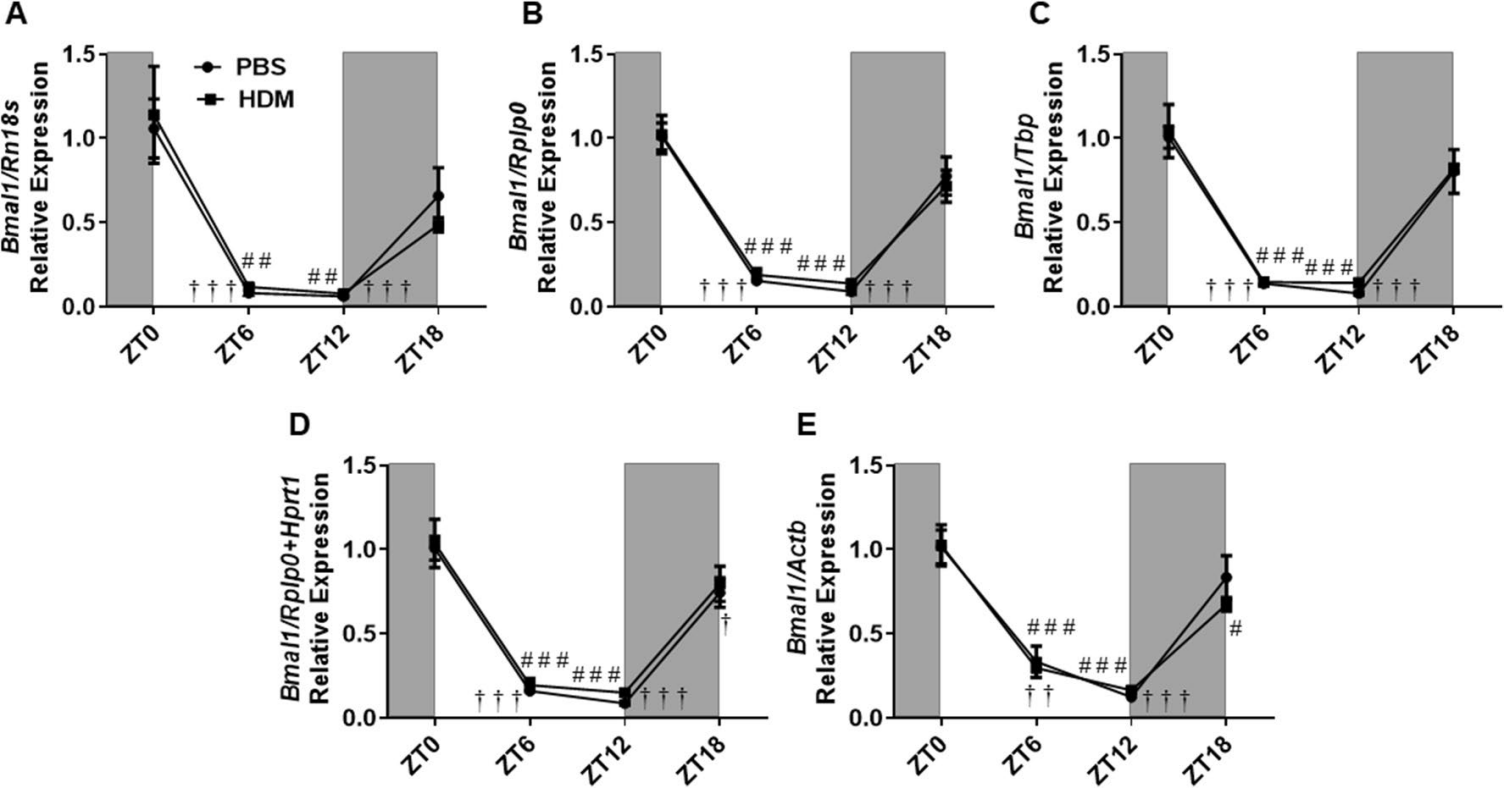
*Bmal1* gene relative expression in HDM and PBS exposed mouse lung and data normalized with different reference genes. (**A**) *Rn18s*, (**B**) *Rplp0*, (**C**) *Tbp*, (**D**) *Rplp0 + Hprt1* and (**E**) *Actb*. Line graphs showing the relative expression of the *Bmal1* gene in both HDM versus PBS group. White and gray areas in the graph represents the light and dark cycle, respectively. Symbols † and # represent the statistical significance in temporal expression of *Bmal1* in the PBS and HDM group respectively at the marked time point when compared to the baseline (ZT0). Data are shown as mean ± SEM (n = 4/group; † *P* < 0.05, †† *P* < 0.01, ††† *P* < 0.001, compared to ZT0 PBS; # *P* < 0.05, # # *P* < 0.01, # # # *P* < 0.001, compared to ZT0 HDM).

The circadian gene, *Bmal1* showed no changes, at least during the acute HDM-sensitization phase in our mouse model. No statistically significant changes were observed between the HDM and the PBS control group. Surprisingly, the relative expression pattern of *Bmal1* also did not change even when normalized using the most stable and the least stable reference genes (Fig. 5). Similarly, *Bmal1* showed no trend in amplitude changes between the PBS and HDM groups (Supplementary Table S1). Temporal variation analysis revealed a significant decrease in the expression of *Bmal1* at ZT6 and ZT12 when compared to the baseline at ZT0 in both PBS and HDM group when normalization was performed using the stable reference genes (*Rn18s, Rplp0, Tbp, Hprt1),* gene combination *(Rplp0* + *Hprt1*) and the least stable reference gene, *Actb*. We also found a significant decrease in *Bmal1* expression at ZT18 versus ZT0 in the PBS when *Hprt1* and *Rplp0* + *Hprt1* were used for normalization (Fig. 5 and Supplementary Figure S1).

**Figure 6:**
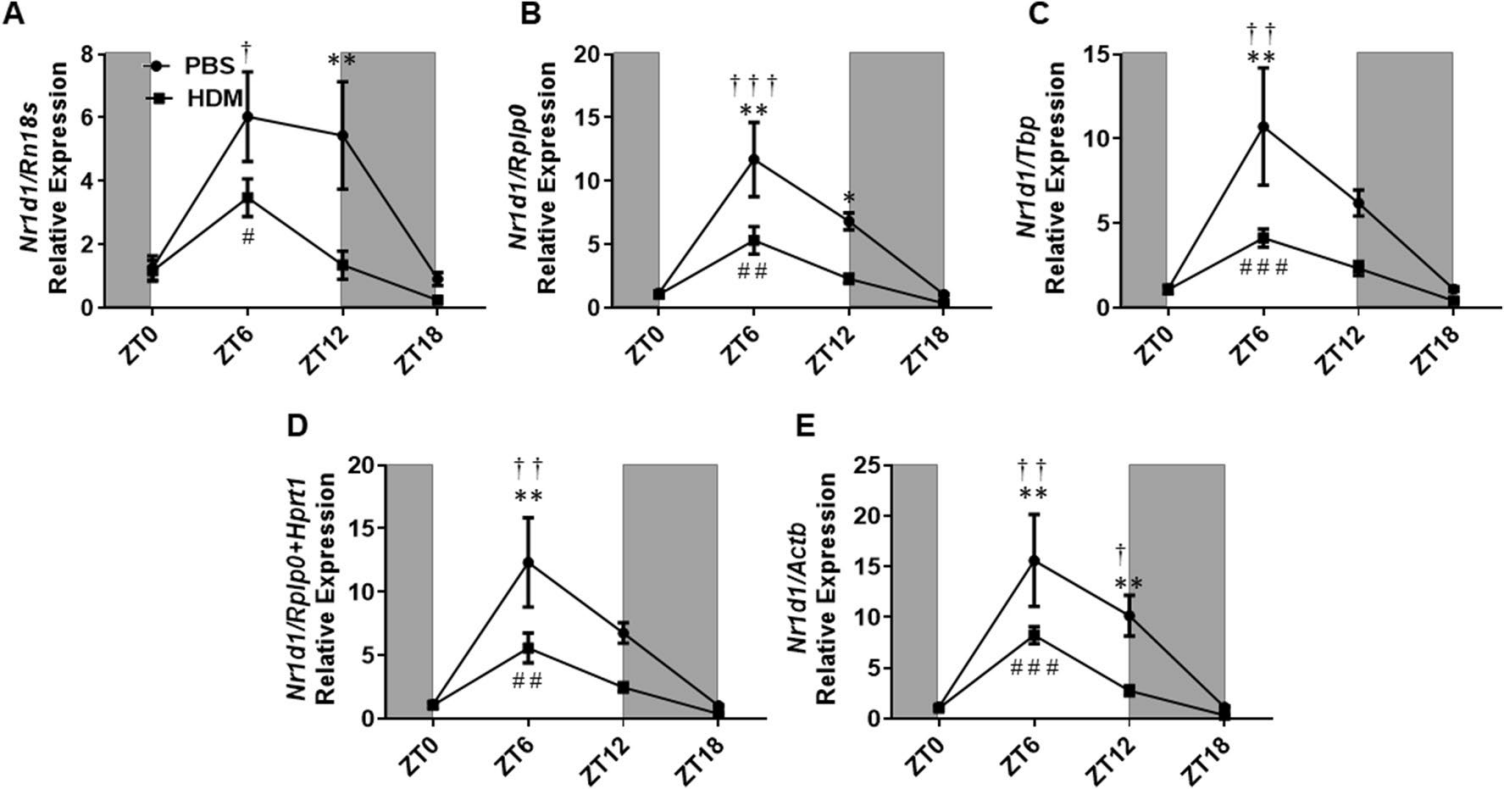
*Nr1d1* gene relative expression in HDM and PBS exposed mouse lungs and data normalized with different reference genes. (**A**) *Rn18s*, (**B**) *Rplp0*, (**C**) *Tbp*, (**D**) *Rplp0 + Hprt1, and* (**E**) *Actb*. Line graphs showing the relative expression of the *Nr1d1* gene in both HDM versus PBS group. White and gray areas in the graph represents the light and dark cycle, respectively. Symbols † and # represent the statistical significance in temporal expression of *Nr1d1* in the PBS and HDM group respectively at the marked time point when compared to the baseline (ZT0). Data are shown as mean ± SEM (n = 4/group; * *P* < 0.05, ** *P* < 0.01, *** *P* < 0.001 compared to respective control; † *P* < 0.05, †† *P* < 0.01, ††† *P* < 0.001, compared to ZT0 PBS; # *P* < 0.05, # # *P* < 0.01, # # # *P* < 0.001, compared to ZT0 HDM).

Circadian gene, *Nr1d1* showed a general trend in decreased relative expression following the HDM exposure during the acute sensitization phase compared to the PBS control group. The amplitude effect in the HDM group also decreased compared to the PBS group when normalization of *Nr1d1* was performed with *Rplp0*, *Tbp,* and *Rplp0* + *Hprt1*. However, the amplitude slightly increased in the HDM group when normalization was performed using *Rn18s* (Supplementary Table S1). Statistical significance in the relative expression of *Nr1d1* between the HDM and PBS group was observed only at ZT6 using *Rplp0, Tbp, Hprt1, Rplp0* + *Hprt1,* and *Actb,* and at ZT12 using *Rn18s*, *Rplp0,* and *Actb* (Fig. 6 and Supplementary Figure S1)*.* Temporal variation analysis revealed a significant increase in the expression of *Nr1d1* at ZT6 versus ZT0 in the PBS and HDM group when normalization was performed using all the stable reference genes (*Rn18s, Rplp0, Tbp, Hprt1*), gene combination (*Rplp0* + *Hprt1*) and the least stable reference gene (*Actb*). However, the only exception was in the PBS group showed a significant increase in the expression of *Nr1d1* at ZT12 versus ZT0 when *Actb* was used for normalization (Fig. 6 and Supplementary Figure S1).

**Figure 7:**
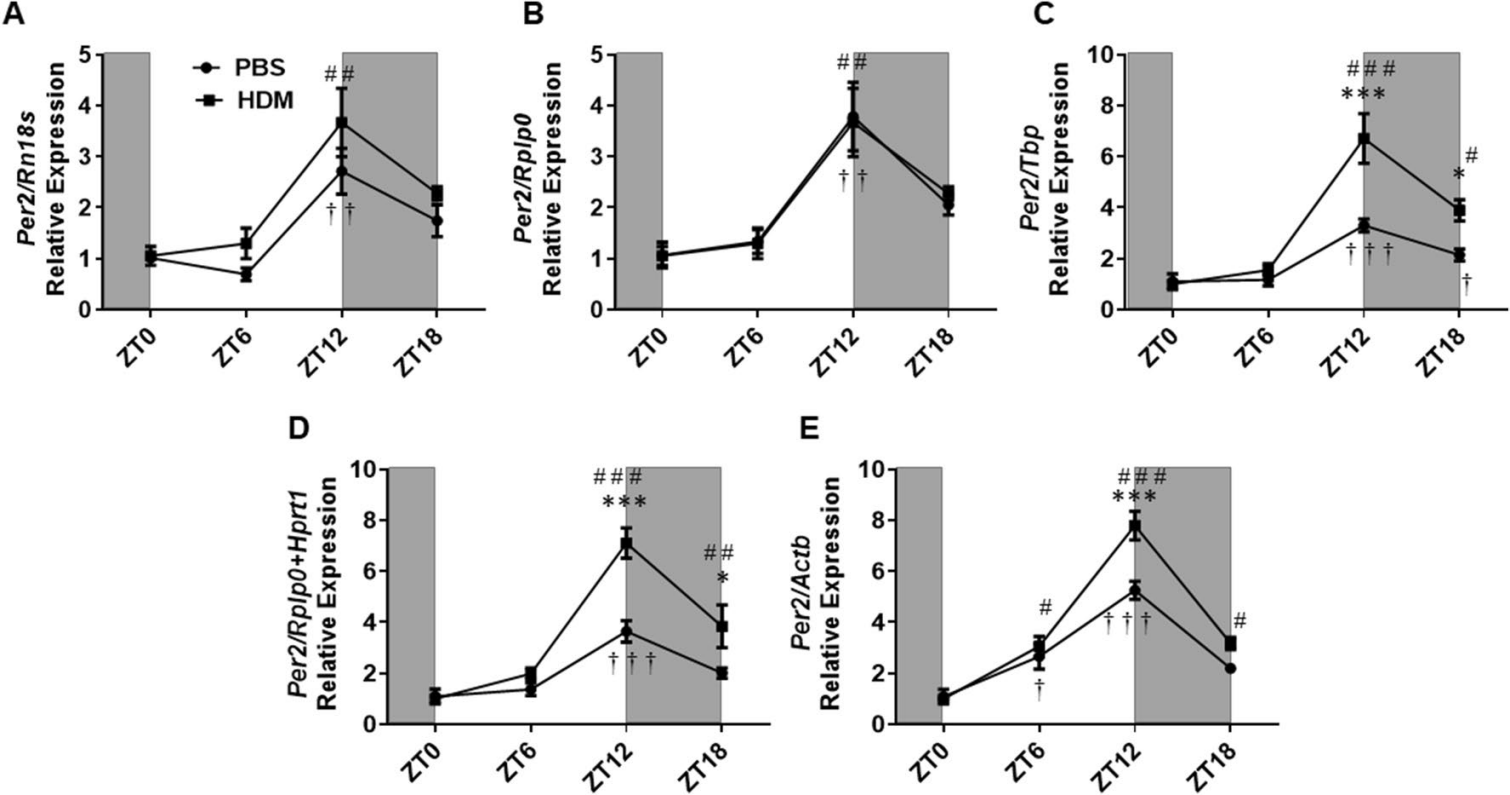
*Per2* gene relative expression in HDM and PBS exposed mouse lungs and data normalized with different reference genes. (**A**) *Rn18s*, (**B**) *Rplp0*, (**C**) *Tbp*, (**D**) *Rplp0 + Hprt1,* and (**E**) *Actb*. Line graphs showing the relative expression of *Per2* gene in both HDM versus PBS group. White and gray areas in the graph represents the light and dark cycle, respectively. Symbols † and # represent the statistical significance in temporal expression of *Per2* in the PBS and HDM group respectively at the marked time point when compared to the baseline (ZT0). Data are shown as mean ± SEM (n = 4/group; * *P* < 0.05, *** *P* < 0.001 compared to respective control; † *P* < 0.05, †† *P* < 0.01, ††† *P* < 0.001, compared to ZT0 PBS; # *P* < 0.05, # # *P* < 0.01, # # # *P* < 0.001, compared to ZT0 HDM).

Similar to the circadian *Clock* gene, acute exposure to HDM during the sensitization phase shows a general trend of increased expression of *Per2* compared to the PBS control group. Higher amplitude in the HDM group also verified this finding when normalization was performed with the stable reference genes (Supplementary Table S1). Statistical significance in the relative expression of *Per2* between the HDM and PBS group was only observed at ZT12 using *Tbp, Hprt1, Rplp0* + *Hprt1,* and *Actb* as reference gene, and at ZT18 using *Tbp, Hprt1,* and *Rplp0* + *Hprt1*. No significance was observed using *Rn18s,* the most stable reference gene (Fig. 7 and Supplementary Figure S1). Temporal variation analysis revealed a significant increase in the expression of *Per2* at ZT12 versus ZT0 in both PBS and HDM group when normalization was performed using all the stable reference genes (*Rn18s, Rplp0, Tbp, Hprt1*), gene combination (*Rplp0* + *Hprt1*) and the least stable reference gene (*Actb*). A significant increase in the expression of *Per2* was also observed at ZT18 versus ZT0 in the HDM group using *Tbp, Hprt1, Rplp0* + *Hprt1,* and *Actb,* and at ZT6 versus ZT0 in both the PBS and HDM group when *Actb* was used for normalization (Fig. 7 and Supplementary Figure S1).

**Figure 8:**
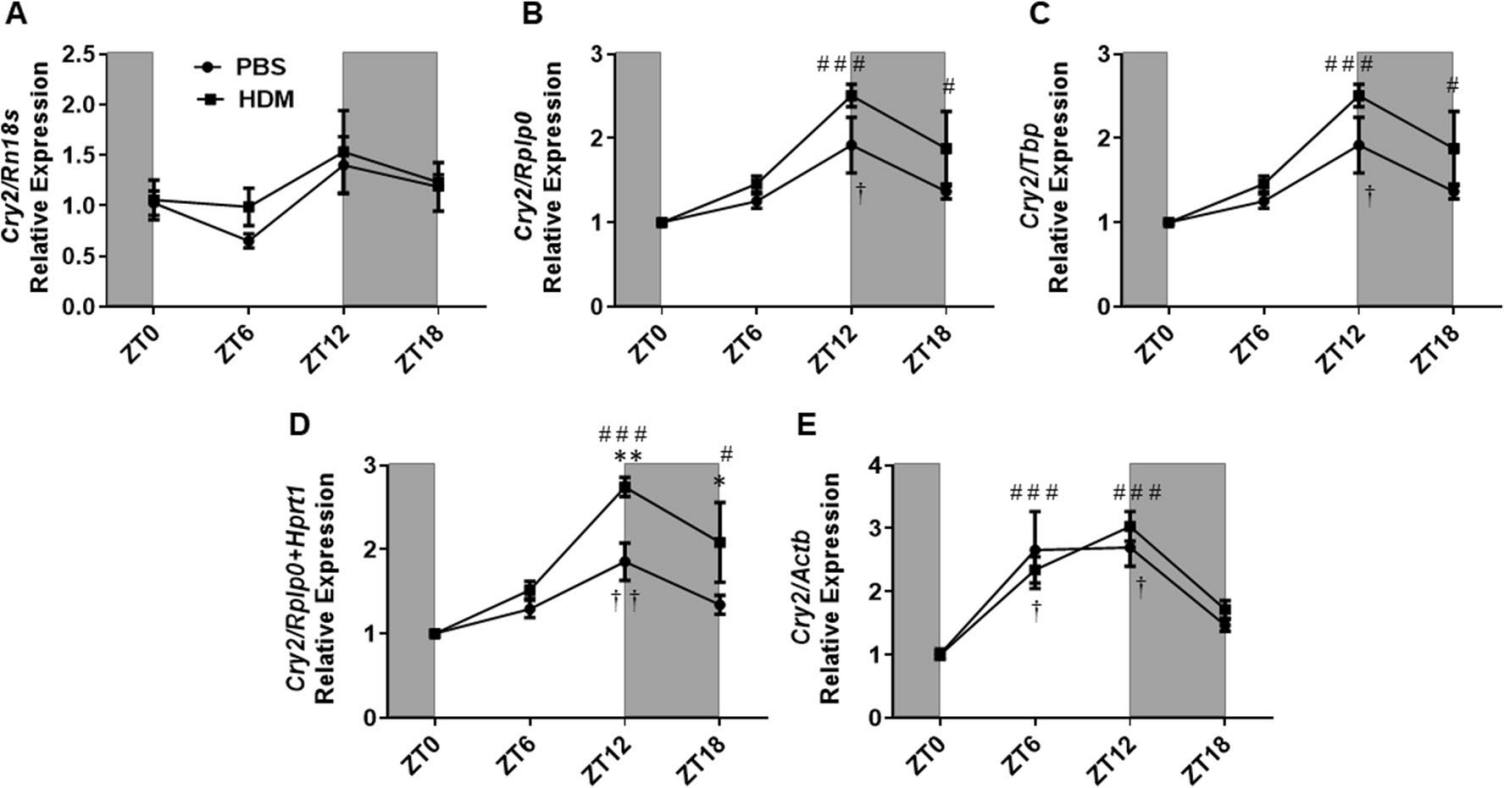
*Cry2* gene relative expression in HDM and PBS exposed mouse lungs and data normalized with different reference genes. (**A**) *Rn18s*, (**B**) *Rplp0*, (**C**) *Tbp*, (**D**) *Rplp0/Hprt1* and (**E**) *Actb*. Line graphs showing the relative expression of *Cry2* gene in both HDM versus PBS group. White and gray areas in the graph represents the light and dark cycle, respectively. Symbols † and # represent the statistical significance in temporal expression of *Cry2* in the PBS and HDM group respectively at the marked time point when compared to the baseline (ZT0). Data are shown as mean ± SEM (n = 4/group; * *P* < 0.05, ** *P* < 0.01 compared to respective control; † *P* < 0.05, †† *P* < 0.01, compared to ZT0 PBS; # *P* < 0.05, # # # *P* < 0.001, compared to ZT0 HDM).

A similar increasing trend in the relative expression was observed for the circadian *Cry2* gene that correlated with increased amplitude in the HDM group compared to PBS when normalization was performed with stable housekeeping genes except for *Rn18s* and *Actb* normalization (Supplementary Table S1). Statistical significance in the relative expression of *Cry2* between the HDM and PBS group was observed only using the *Rplp0* + *Hprt1* and *Hprt1* at ZT12 and ZT18. Normalization with *Actb*, the least stable reference gene offsets the relative expression pattern, showing almost no change in the expression between ZT6 and ZT12 for the PBS group (Fig. 8 and Supplementary Figure S1). Furthermore, we also noticed that normalization with *Actb* reference gene exaggerates the relative expression pattern to a much greater extent even in the PBS group time-dependently than any of the top 3 reference genes evaluated in the study (Supplementary Figure S2, S3). Temporal variation analysis revealed a significant increase in the expression of *Cry2* at ZT12 versus ZT0 in both PBS and HDM groups when normalization was performed using all the stable reference genes (*Rplp0, Tbp, Hprt1*), gene combination (*Rplp0* + *Hprt1*), and the least stable reference gene (*Actb*). However, we did not observe any temporal variation in the expression of *Cry2* in both PBS and HDM groups when *Rn18s* was used for normalization. A significant increase in the expression of *Cry2* was observed at ZT18 versus ZT0 in the HDM group using *Rplp0*, *Tbp, Hprt1,* and *Rplp0* + *Hprt1,* and at ZT6 versus ZT0 in both the PBS and HDM group when *Actb* was used for normalization (Fig. 8 and Supplementary Figure S1).

### CircWave analysis

CircWave analysis further revealed finer details of our data including determining diurnal variation and the peak phase expression of the core CCGs. When the normalization of the relative expression of *Clock* in the PBS (control) group was performed using the top 3 and best combination of the reference gene, CircWave results revealed a strong diurnal expression pattern for *Clock* gene transcription as indicated by the low *p*-value obtained from the analysis. However, when *Actb* the least stable reference gene was used for normalization in the PBS group, the observed diurnal expression pattern was completely abolished. The peak expression level of *Clock* however was very similar in the PBS group as indicated by the center of gravity (COG) values (Supplementary Table S1 and Supplementary Figure S2). Furthermore, when *Clock* gene expression was normalized in the HDM group, a diurnal pattern of expression was detected by CircWave for all the reference genes used, including *Actb*. However, the peak expression of *Clock* when normalized with *Actb* was considerably lower, showing a peak COG as 14.9, than when measured with the top 3 and best combination of reference genes which ranged between 17.5 and 20.9 (Supplementary Table S1).

CircWave analysis did not reveal any extreme changes when normalization with all the reference genes was performed for *Bmal1* and *Nr1d1*, at least not within the same group (HDM or PBS). For instance, *Bmal1* expression in the PBS group showed a peak expression with a COG 21, and this was consistent when the top 3, best combination and least stable reference gene was used for normalization. However, for the HDM group the peak expression of *Bmal1* slightly shifted to the right showing a peak expression with a COG 23 when normalized with *Rn18s* and *Actb*, while with a COG 22 when normalized with *Rplp0, Tbp* and best gene combination (*Rplp0* + *Hprt1*). Similarly, for *Nr1d1*, peak expression in the PBS group was observed around 8.7 when normalized with *Rn18s* and with a COG between 7.2 and 7.9 with *Rplp0, Tbp*, best combination and *Actb*. *Nr1d1* peak expression in the HDM however shifted slightly to the left, showing a peak expression with a COG between 6.2 and 6.9 when normalized with the *Rn18s, Rplp0*, best combination and *Actb*, and with a COG 7.2 using *Tbp*. *Bmal1* and *Nr1d1* expression were determined to be rhythmic in both PBS and HDM groups when normalized with any of the reference genes (Supplementary Table S1).

CircWave analysis of *Per2* expression revealed a strong diurnal variation in both PBS and HDM groups, although the peak expression slightly varied among and between the two groups. For instance, *Per2* expression in the PBS group showed a peak expression with a COG around 14.5 using *Rn18s* and *Tbp*, while the COG was 13.6 using *Rplp0* and best combination. However, when using *Actb* it showed a peak expression with a COG 12.1. Similarly, for the HDM group, the peak expression of *Per2* was observed with a COG 12.5 when normalization was performed with the top 3 and the best combination reference genes, while the peak expression was observed with a COG 11.6 when normalized with *Actb* (Supplementary Table S1).

Interestingly, a diurnal variation in the expression pattern for *Cry2* was not detected by CircWave in either PBS or HDM group when normalization was performed using *Rn18s*, the most stable reference gene determined in our study. However, normalization with the other reference genes including *Rplp0, Tbp*, best combination and *Actb*, were found to be rhythmic. When normalization was performed using *Rn18s*, the peak expression of *Cry2* in the PBS group was found with a COG 16.4, and around 13.6 when normalized with *Rplp0*, best combination and *Tbp*. In the HDM group, a similar pattern was observed for *Cry2*. While normalization with *Rn18s* and *Rplp0* revealed the peak expression with a COG 11.6, and between 12.1 and 12.9 with *Tbp* and best combination, *Actb* showed the peak expression with a COG 10.2. The exact COG determined by CircWave analysis for all the CCGs from PBS and HDM groups, the data mean and their *P*-values with *r*^*2*^ is summarized (Supplementary Table S1).

### Temporal variation in the expression of the reference genes

CircWave analysis revealed that the most stable reference genes (*Rn18s, Rplp0,* and *Tbp*) showed no diurnal variation in the PBS group when normalization was performed using the most stable and the best combination (*Rplp0* + *Hprt1*) of the reference genes. These findings were also true in the HDM group for *Rn18s* when normalization was performed with the most stable and best combination of reference genes. When the reference genes *Rplp0* and *Tbp* in the HDM group were normalized with *Rn18s*, and the best combination of reference gene no diurnal variation was detected by CircWave. However, when *Rplp0* and *Tbp* were normalized with *Tbp* and *Rplp0* respectively, a diurnal expression pattern was detected by CircWave in the HDM group. Not surprisingly, a diurnal variation was detected for all instances by CircWave analysis when the least stable reference gene *Actb* was normalized with the most stable and the best combination of reference genes in both PBS and HDM group (Supplementary Table S2 and Supplementary Figure S4A,B).

## Discussion

Circadian rhythms have an established role in health and disease and the lung is perhaps one of the vital peripheral organs whose functions are greatly influenced by it^2^. Thus, basic and clinical translational researchers utilize acute and chronic lung injury models to understand the novel molecular mechanisms regulated by the circadian clock. Circadian studies are conducted in mouse models where cell-type and tissue-specific changes in circadian clock gene expression are evaluated. Prior studies have demonstrated circadian clock disruption in the lung using acute or chronic exposure to different environmental agents (e.g., environmental tobacco smoke/cigarette smoke, pollutants, house dust mite allergen and ozone, etc.), bacteria or viruses (e.g., LPS, Influenza A virus) and chemicals (e.g., bleomycin), that causes pulmonary diseases^5,18–20^. This study is focused to determine the most stable reference gene for lung circadian clock studies to evaluate time-dependent change in clock gene expression in mice.

In this study, lung tissues from an acute HDM-sensitization model were used to evaluated circadian clock gene expression. We tested the 10 most commonly used reference genes for normalization and evaluate their stability using five unique algorithms (GeNorm, NormFinder, BestKeeper, RefFinder and Qbase+). It was interesting to note that all the four algorithms determined the same, top 4 or 5 stable reference genes as the best reference genes to use for normalization, except that the ranking slightly varied. For instance, *Rn18s* was ranked as the most stable reference gene using NormFinder, BestKeeper and RefFinder, while second most stable reference gene according to GeNorm. Similarly, *Actb* was ranked as the least stable reference gene according to GeNorm, NormFinder and RefFinder, but third last according to BestKeeper.

To examine how the gene expression levels of core CCGs change when normalized with different reference genes, we selected the top 3 stable reference genes (*Rn18s, Rplp0, Tbp*) and the least stable reference gene (*Actb*) determined by RefFinder, and the best combination of stable genes (*Rplp0* + *Hprt1*) determined by GeNorm. We normalized the Cq values of Target gene (CCGs)—Cq values of stable reference gene using the 2^-ΔΔCt^ method with selected reference genes identified in this study. The relative expression of the core CCGs was relatively similar when normalized with *Rn18s, Rplp0, Tbp* and *Rplp0* + *Hprt1*. When normalization was performed with the least stable reference gene *Actb*, in almost all target genes analyzed, an exaggerated relative fold change was observed in the PBS group for time-dependent change in *Clock* gene expression in the lung. The circadian *Clock* gene showed a diurnal expression pattern in the mouse lung. For time-dependent change in gene expression rhythms of CCGs in the mouse lung, we referred to the CircaDB lung microarray data (http://circadb.hogeneschlab.org/mouse)^21^. CircWave analysis revealed that this diurnal expression pattern holds true when the most stable reference genes, including the *Rplp0* + *Hprt1* is used for normalization. However, when normalization was performed using *Actb,* the diurnal expression pattern of the *Clock* gene was completely abolished. Additionally, CircWave also revealed how exposure to HDM alters the peak expression (COG) of *Clock* gene when normalized with *Actb* (showing a peak with a COG 14.8) while remaining relatively constant for the stable reference genes (showing peak expression with a COG between 17.5 and 20.9). Temporal variation analysis of *Clock* gene expression in the PBS group revealed a significant decrease at ZT6 versus ZT0 when the most stable (*Rn18s, Tbp, Rplp0*) and best combination (*Rplp0* + *Hprt1*) reference genes were used for normalization. However, when the normalization was performed using *Actb* no diurnal variation in *Clock* gene expression was observed. Interestingly, when we analyzed the expression of *Clock* in the PBS group starting from the most stable and moving to the least stable reference genes identified in this study, the more significant diurnal changes were observed. For instance, when we used the most stable (*Rn18s* and *Rplp0*) and the best combination (*Rplp0* + *Hprt1*) reference genes for normalization of the *Clock* gene in the PBS group a significant reduction was observed only at ZT6 versus ZT0. However, when we used the top third (*Tbp*) and the top fourth (*Hprt1*) reference genes for normalization, we found a significant difference at both ZT6 and ZT12 versus ZT0.

Similarly, *Cry2* expression is also known to peak around ZT12 as per data shown in CircaDB^21^, and this supports our findings only when normalized with the top 3 or best combination of the reference genes (*Rn18s, Rplp0, Tbp* and *Rplp0* + *Hprt1*). Normalization with *Actb* shows an expression pattern very different from what we obtained by normalizing with other references genes. Uncertainty in measurement using *Actb* was so high that no relative difference in expression could be observed at ZT6 versus ZT12 for *Cry2* even in the control group. CircWave also confirmed our findings revealing different COG values when normalization is performed using *Actb*, compared to the other reference genes. A similar observation was also made for *Per2* and *Clock* genes in both HDM and PBS groups. Additionally, when we evaluated the temporal variation in expression of *Per2* and *Cry2*, we noticed that although normalization with *Actb* showed a significant difference in the expression pattern at ZT6 versus ZT0 in both PBS and HDM groups. However, normalization with the most stable (*Rn18s, Tbp, Rplp0*) and best combination (*Rplp0* + *Hprt1*) reference genes revealed no significant difference between ZT6 versus ZT0 in both PBS and HDM groups. These findings further highlight that the use of *Actb* might not be appropriate for normalizing CCGs to conduct relative expression as well as temporal expression analyses.

In general, relative expression analysis of the core CCGs between the HDM and PBS group revealed that *Clock, Per2 and Cry2* gene expression tend to increase, while *Nr1d1* gene expression tends to decrease following acute HDM-sensitization compared to the PBS control. This was true even when data normalization was performed for all core CCGs using the least stable reference gene (*Actb),* except for *Cry2*. No change in the expression of *Bmal1* in the HDM versus PBS exposed group was observed. CircWave analysis was utilized to evaluate the change in amplitude between the HDM and PBS group, and the results corroborated with similar changes (increased relative expression corresponding to higher amplitude and decreased relative expression corresponding to decrease in amplitude) when normalization was performed using the stable housekeeping genes. Interestingly, with the only exception of the relative expression of *Nr1d1* at ZT12, when *Rn18s*, the most stable reference gene was used for normalization of the data, no other time points were found to be significant for *Nr1d1*. Similarly, neither of the other core CCGs including *Clock, Bmal1, Per2* and *Cry2* measured at four different time points (ZT0, ZT6, ZT12, ZT18) showed any significant difference between the HDM and PBS groups using *Rn18s* as the reference gene. However, strong statistical significance was observed at ZT6 when *Nr1d1* was normalized with reference gene *Rplp0, Tbp, Hprt1, Actb* and *Rplp0* + *Hprt1*, and at ZT12 using *Rplp0* and *Actb*. Similarly, *Clock* showed a significant difference at ZT18 when normalized with *Rplp0, Tbp, Hprt1,* and *Rplp0* + *Hprt1*, and at ZT12 using *Tbp* and *Hprt1*. *Per2* also showed a significant difference at ZT12 when normalized with *Tbp, Hprt1, Actb* and *Rplp0* + *Hprt1*, and *Cry2* showed statistical significance at ZT12 and ZT18 when normalized with *Hprt1* and *Rplp0* + *Hprt1*. A plausible explanation behind this discrepancy could be the use of an acute HDM-sensitization model. Acute HDM-sensitization (single dose) may not have a strong impact on the expression of core CCGs between the HDM and PBS control groups as short as 6 h post-treatment. Therefore, we believe that the use of the *Rn18s* may be more appropriate, and the normalized data with *Rn18s* showing no significant changes between the HDM treated and PBS control group, is probably a true reflection of our findings. Furthermore, looking at the narrow difference in Cq values for *Rn18s* across all samples analyzed, we realize that *Rn18s* is undoubtedly the most stable reference gene that was neither affected by the treatment conditions in the mouse lung.

The relative expression of the core CCGs in the HDM group was relatively similar when normalized with the top 3 (*Rn18s, Rplp0, Tbp*), best combination (*Rplp0* + *Hprt1*) reference genes but not when normalized with *Actb*. For instance, the temporal expression of *Clock* in the HDM group showed a significant decrease at ZT6 versus ZT0 when normalization was performed using most stable reference genes *Rn18s, Rplp0* and *Tbp*, but not with *Actb.* Likewise, *Bmal1* also showed a significant decrease at ZT18 versus ZT0 in the HDM group when *Actb* was used for normalization but not when the top 3 stable reference genes were used. Similarly, there was no significant difference in the temporal expression pattern of *Per2* in the HDM group at ZT6 versus ZT0 when using *Rn18s, Rplp0, Tbp,* and *Rplp0* + *Hprt1*, but normalization with *Actb* showed a significant difference. Similar outcomes were observed for the temporal expression pattern of *Cry2* in PBS and HDM group at ZT6 versus ZT0 when normalization was performed using *Actb.* We observed that when normalization was performed using *Rn18s*, the relative expression was consistently lower for all the CCGs analyzed compared to the other reference genes. Overall, as mentioned earlier, normalization with *Rn18s* could be a better reflection of the actual relative expression and temporal variation of the CCGs in the lungs of PBS and HDM groups.

CircaDB provides compiled data of known rhythmic patterns and peak expression of the core CCGs observed in mouse tissues^21^. From CircaDB, we know the peak expression of the core CCGs evaluated in our study including *Clock* (ZT20-22)*, Bmal1* (ZT 21–23)*, Nr1d1* (ZT5-7)*, Per2* (ZT11-13) and *Cry2* (ZT9-11). When *Rn18s*, the most stable reference gene was used for normalization, CircWave analysis found the peak expression at ZT20.2, ZT21.5, ZT8.7, ZT14.7 and ZT16.3 in the PBS group and ZT20.9, ZT23.1, ZT6.2, ZT12.6, and ZT11.4 in the HDM group for *Clock, Bmal1, Nr1d1, Per2* and *Cry2* respectively. However, when the least unstable reference gene, *Actb*, was used for normalization the peak expression was found at ZT19.1, ZT21.4, ZT7.2, ZT12.1, ZT10.3 in the PBS group and ZT14.8, ZT23.1, ZT6.8, ZT11.6, ZT10.2 in the HDM group for *Clock, Bmal1, Nr1d1, Per2* and *Cry2* respectively. The rhythmic expression of *Cry2* in the PBS group and *Clock* in the HDM group was skewed when *Actb* was used for normalization (compared to *Rn18s*). As defined earlier, a stable reference gene should neither vary with time nor treatment or exposure (e.g., HDM). Our study provides strong evidence for the first time that there is an observed diurnal variation in the expression of *Actb* in PBS and HDM exposed mouse lungs. Thus, *Actb* should not be used for normalization in a qRT-PCR analysis of mouse lungs that depend on the agents/chemicals that affect the gene expression of cytoskeletal targets.

In the past few years, our knowledge of the core CCGs and how it contributes to the circadian aspects of sleep, metabolism, physiology, regulation of immune function, etc., has greatly improved. This had triggered interest among scientists to use circadian clock gene knockout mouse models to further understand circadian biology in health and disease. In our study, we only utilized wild-type (WT) mice. Although inconclusive from our study, we speculate that the stability of the reference gene in the lung might not vary among different transgenic strains and clock gene knockout mice, at least not when the same agent is used to induce lung injury. Additionally, we tested the stability of the reference gene in the mouse lung using an acute HDM-sensitization model, in which mice were exposed to HDM or PBS post 6 h before they were euthanized. This would allow us to determine if there is any significant change in the expression level of the core CCGs following HDM exposure during the sensitization phase as early as 6 h post-exposure. Thus, how the stability of the reference gene changes following exposure to HDM in a chronic model remains unclear. Furthermore, we only included lung tissues from 4 different time points (ZT0, ZT6, ZT12 and ZT18) in our study. We believe that additional time points to measure the relative expression patterns every 4 h instead of 6 h, and for a period of 24–48 h would provide us with a deeper understanding of how the rhythmic expression of CCGs changes over time.

Previously, Gibbs et al. utilized CCSP-^icre+/-^ and Bmal1^fl/fl^ mice to determine clock gene expression by qRT-PCR analysis over 24 h (every 6 h) in the lung and liver using *Actb* and *36B4* as HKG. They found no significant difference in the rhythmic expression of CCGs (*Per2, Bmal1, Rev-erbα*, and *Cry1*) between the genotypes analyzed in the lung. In the same study, they utilized LPS and *Streptococcus pneumoniae* infection models to demonstrate time-dependent circadian regulation of chemokines in lung inflammation and glucocorticoid action using the above-mentioned HKG without evaluating the effects on reference genes appropriately for different models^6^. Another study from the same group showed the qRT-PCR analysis of chemokines and CCGs measured at two different time points or different ZT’s (every 4 h) analyzed from lung tissues or synchronized cells normalized with *Rn18s* as HKG^7^. We have previously used both *Rn18s* and *Gapdh* for acute and chronic circadian studies using environmental tobacco smoke exposures to determine gene expression of cytokines and CCGs in the lungs of WT and *Rev-erbα* KO mice^5,11^. Another report using hyperoxia model, evaluate the rhythmic expression of CCGs in WT mice (6 h interval) and pro-inflammatory genes (at ZT11 vs. ZT23) in the lungs of alveolar type II cell-specific Bmal1 KO was analyzed using *Rn18s* and *28S* as HKG^8^. Other studies from the hypoxia model utilized geometric mean of two or three HKG (*Rplp0* + *Tbp* or *Tbp* + *Hprt* + *Rplp0*) without clearly indicating how they decided to utilize these combinations for qRT-PCR analysis to evaluate clock-associated genes in a time- and tissue-specific manner^9,10^. These studies demonstrate the need for a thorough evaluation of stable reference genes for the lung injury models before conducting time- and tissue-specific analysis of circadian CCGs as well as other related canonical pathway-associated genes involved in the study. It may be vital for the researchers conducting circadian clock studies using different models to understand the role of circadian clock targets in the pathobiology of chronic inflammatory diseases. Researchers in the field should consistently utilize the validated stable reference gene or HKG for their ongoing and future studies for better comparison and correct interpretation of the findings.

Overall, our results demonstrate that choosing a stable reference gene is crucial for normalization in a qRT-PCR assay or it can result in misinterpretation of the data. Here, we found *Rn18s* to be the most stable reference gene, and *Actb* as the least stable reference gene for normalization in acute HDM-sensitization using mouse lungs. This is in line with another recent study that revealed *Actb* as the least stable reference gene for circadian studies in mouse liver and adrenal gland in different mouse strains^22^. In this study, we systematically show how the use of *Actb* often leads to exaggerated relative fold changes between the PBS and HDM groups, or even completely abolish the known rhythmic pattern that is shown previously. CircWave analysis further revealed how peak expression of the CCGs change when the least stable reference gene like *Actb* are used for normalization. Based on our findings, we recommend the use of the *Rn18s* as the go-to reference gene for qRT-PCR normalization in circadian studies involving the mouse lungs.

## Materials and methods

### Experimental animals

Eight weeks old C57BL/6 J (wild-type, WT) male and female mice were purchased from Jackson Laboratory (Jax mice, Bar Harbor, ME) for our study. Mice were housed in a controlled environment with regular 12:12-h light:dark cycle (light on at 6:00 am (Zeitgeber Time—ZT0) and light off at 6:00 pm (Zeitgeber Time—ZT12) with *ab libitum* access to food and water. All animal experiments were performed as per the approved protocol by the Institutional Animal Care and Use Committee (IACUC) at the University of Kansas Medical Center (ACUP # 2020–2575), and the National Institute of Health (NIH) Guide for Care and Use of Laboratory Animals. The animal experiments described in this study were conducted in accordance with ARRIVE guidelines.

### Acute HDM-sensitization model

A total of 33 mice were utilized for the study. First, mice were randomly assigned into two groups: one that was sensitized with house dust mite (HDM) and the other with Phosphate-Buffered Saline (PBS), the control group. Then, each of the two groups was further subdivided into 4 groups: ZT0, ZT6, ZT12 and ZT18, which corresponded to 6:00 am, 12:00 pm, 6:00 pm and 12:00 am respectively of the regular 12:12 light–dark cycle. Care was taken to ensure that each group had at least 4 mice in each time point (ZT0, ZT6, ZT12 and ZT18). Mice from each group were dosed with either PBS or HDM 30 μg (30 μl) intranasally at ZT18, ZT0, ZT6, and ZT12 under light isoflurane anesthesia, and lung tissues were harvested every 6 h post-treatment at ZT0, ZT6, ZT12, and ZT18 respectively. Lung lobes were immediately snap-frozen in liquid nitrogen and stored at − 80 °C for later analysis.

### Total RNA extraction and cDNA preparation

Lung samples were thawed in ice, homogenized in 700 µl of QIAzol lysis reagent and RNA was extracted using the miRNeasy kit (Qiagen, Valencia, CA) according to the manufacturer’s protocol. Additionally, DNase treatment was performed using DNase I (Zymo Research, USA) for each sample. Following RNA extraction, the concentration and purity were determined by a Nanodrop spectrophotometer (Thermo Scientific, Wilmington, DE). cDNA synthesis was performed using iScript cDNA Synthesis Kit (Bio-Rad, USA). 1 μg of RNA was mixed with 4 µl of 5 × iScript reaction mix, 1 µl of iScript reverse transcriptase, 2 µl of random primers, and a variable amount of RNAse free water to make the final volume to 20 µl. The mixture was incubated in a thermal cycler at 25 °C for 5 min, 46 °C for 20 min, 95 °C for 1 min and held at 4 °C. Following cDNA synthesis 40 µl of RNAse free water was added to make the final volume to 60 µl.

### Quantitative real-time polymerase chain reaction (qRT-PCR) analysis

All the qRT-PCR reactions were performed using iTaq Universal SYBR Green Supermix *(*Bio-Rad*)* and gene-specific primers (circadian clock genes and reference genes) using the CFX Opus 96 Real-time PCR System (Bio-Rad). Relative expression of target genes was determined by the 2^-ΔΔCt^ method with top 4 most stable reference genes or their combinations (*Rn18s, Rplp0, Tbp, Hprt1 and Rplp0* + *Hprt1*) and the least stable reference gene, *Actb* as described previously. The quantification cycle (Cq) value used for the best combination is the geometric mean of the Cq value of the two individual reference genes. Mouse circadian gene-specific primers (*Clock, Bmal1, Nr1d1, Per2,* and *Cry2*) and 10 different reference genes were synthesized by IDT (www.idtdna.com). Primer sequences for the circadian genes and reference genes evaluated in the study were either obtained from published literature or real-time PCR primer database (PrimerBank https://pga.mgh.harvard.edu/primerbank/) as summarized in Table 1.

### Primer efficiency

The primer efficiencies were calculated by generating a standard curve for each target gene using a five-fold serial dilution of the cDNA pool, and using the slope to calculate the efficiency using the formula E = 10^(-1/Slope)^ as described previously^23^.

### Analysis of expression stability

Five different statistical algorithms including GeNorm, NormFinder, BestKeeper, RefFinder and Qbase+ were used to evaluate the expression stability of the 10 reference genes used in our study. GeNorm identifies the most stable reference genes based on the pairwise variation among the samples and then generates a stability (M) value following a stepwise exclusion of the least stable reference gene. The lower the M value, the higher the stability. An added advantage of GeNorm is that it also determines the optimal number of reference genes needed for accurate normalization^16^. We used the GeNorm algorithm in two different software using an online tool known as RefFinder^13^, and a commercially available software, version 3.0 Qbase + (Biogazelle, Zwijnaarde, Belgium—www.qbaseplus.com).

The basis of using the two different GeNorm software was to calculate the stability values of the individual reference genes as well as the best combination of a reference gene that can be used for normalization. Qbase + was used to calculate the stability values for individual reference genes and the optimal number of reference genes needed for accurate normalization. Qbase + is unable to give us the best combination of reference genes. RefFinder on the other hand provides us with the best gene combination that can be used for accurate normalization. Similar to GeNorm, NormFinder also gives us a stability value by performing a grouped analysis of the candidate reference genes. The lower the stability value obtained by NormFinder, the greater is the stability of the reference genes^15^. BestKeeper assumes that the higher the standard deviation (SD) or CV values, the less stable the reference genes. A reference gene with a SD value of more than 1 is generally considered unstable and not appropriate for normalization. Lower the SD, greater the stability^14^. Finally, we used RefFinder to give us the comprehensive ranking which combines the output of all the three algorithms as well as delta-CT (not evaluated in our study)^13^. This RefFinder analysis of the most stable and least stable reference gene was used for the reference gene validation described below.

### Reference gene validation

The top four most stable and the least stable reference genes identified by RefFinder, and the best combination of reference genes identified by GeNorm were taken into consideration when normalizing the relative expression of core circadian genes *Clock, Bmal1, Nr1d1, Per2,* and *Cry2* analyzed in this study. Each of the individual reference genes were normalized with all the five selected circadian genes from both the experimental groups (PBS and HDM) and at all the Zeitgeber time points (ZT0, ZT6, ZT12, and ZT18). For simplicity, the qRT-PCR data analysis of the fourth most stable reference gene, *Hprt1*, has been moved to the supplement section of the article.

### CircWave analysis

The diurnal rhythmicity in the gene expression level of the core CCGs were analyzed using CircWave (software version 1.4). CircWave employs a linear harmonic regression model to empirically determine the profile of the input data by adding harmonics to the principal wave function. *P*-values are given as false discovery rate (FDR) corrected. CCGs with an FDR value of less than 0.05 were considered rhythmic^24,25^. An added advantage of using CircWave is that it determines the center of gravity (COG), or the peak phase of the rhythm^25^. This will allow us to visualize how the peak expression of CCGs varies when normalization is performed using different reference genes. Amplitude was determined as percentage of the data mean using the formula ([difference between the highest point – lowest point]/ Data mean*100%) as described previously^26^.

### Temporal variation of the reference genes using CircWave

To determine the temporal variation of the reference genes throughout the day (Zeitgeber time points: ZT0, ZT6, ZT12 and ZT18), we selected the top 3 most stable (*Rn18s*, *Rplp0* and *Tbp*) and least stable (*Actb*) reference genes determined by RefFinder. Then, we normalized each of the selected reference genes with the other stable reference genes evaluated in our study, including the best combination of reference genes (*Rplp0* + *Hprt1*). Having determined the temporal variation of the reference genes, we performed the CircWave analysis to determine if the selected reference genes show a diurnal expression pattern in the mouse lung.

### Statistical analysis

Statistical differences in the relative expression of the clock-controlled genes at different Zeitgeber time points (ZT0, ZT6, ZT12 and ZT18) between PBS and HDM groups were analyzed by two-way ANOVA (using Tukey's multiple-comparison test). Additionally, a significant difference in temporal variation in the expression of CCGs in the PBS and HDM group was determined by comparing the relative expression of the CCGs between the different zeitgeber time points (ZT6, ZT12, and ZT18) to the baseline expression at ZT0 in PBS and HDM group separately, and analyzed the data using one-way ANOVA (using Tukey’s multiple-comparison test). All the above-mentioned statistical analyses were conducted using the GraphPad Prism 9 software (La Jolla, CA). The results are shown as means ± SEM with a *P* < 0.05 considered as statistically significant.

## Supplementary Information

Supplementary Tables.

Supplementary Figures.
