# Supplementary material for: Evaluation of stable reference genes for qPCR normalization in circadian studies related to lung inflammation and injury in mouse model
Source: Sci Rep. 2022 Feb 2;12:1764. doi: 10.1038/s41598-022-05836-1 (PMC8810972; doi:10.1038/s41598-022-05836-1)
Supplement: Supplementary file 1 — Supplementary Tables. [file 41598_2022_5836_MOESM1_ESM.pdf]

Supplementary Table S1. CircWave analysis showing rhythmic expression of circadian clock gene expression in the lungs

Circadian Gene(s)	Housekeeping Gene(s)	Treatment Group	Amplitude (% of Data Mean)	Center of Gravity (COG) ± SD	Data Mean ± SD	*P*-value	r^2
Clock	*Rn18s*	PBS	101.67	20.231 ± 2.361	0.687 ± 0.476	0.008	0.4907
		HDM	91.64	20.966 ± 3.027	0.779 ± 0.375	0.002	0.6428
	Rplp0	PBS	81.93	19.499 ± 2.699	0.797 ± 0.352	0.001	0.6104
		HDM	101.49	17.984 ± 3.198	1.042 ± 0.456	0.002	0.6344
	Tbp	PBS	96.34	19.676 ± 2.511	0.775 ± 0.378	8.7E-05	0.7369
		HDM	135.07	17.979 ± 2.908	1.050 ± 0.513	1E-06	0.9038
	Rplp0+Hprt1	PBS	78.69	19.709 ± 2.708	0.784 ± 0.331	0.0004	0.6694
		HDM	109.42	17.492 ± 3.129	1.112 ± 0.515	0.001	0.6591
	Actb	PBS	34.11	19.126 ± 3.222	0.971 ± 0.296	NaN	-0.2282
		HDM	49.67	14.829 ± 3.577	1.166 ± 0.272	0.004	0.5894
Bmal1	*Rn18s*	PBS	209.09	21.563 ± 1.480	0.475 ± 0.484	0.001	0.7099
		HDM	234.38	23.125 ± 1.368	0.453 ± 0.528	0.001	0.654
	Rplp0	PBS	178.01	21.236 ± 1.708	0.524 ± 0.426	1E-06	0.8722
		HDM	175.09	22.567 ± 1.902	0.504 ± 0.410	3E-06	0.8785
	Tbp	PBS	177.24	21.138 ± 1.644	0.524 ± 0.441	1E-06	0.8655
		HDM	173.30	22.170 ± 1.830	0.519 ± 0.447	4E-06	0.8717
	Rplp0+Hprt1	PBS	179.43	21.337 ± 1.716	0.514 ± 0.410	0	0.9054
		HDM	167.19	22.378 ± 1.950	0.529 ± 0.429	8E-06	0.858
	Actb	PBS	150.82	21.439 ± 2.089	0.591 ± 0.411	1.5E-05	0.7955
		HDM	161.65	23.106 ± 2.126	0.532 ± 0.379	6E-06	0.8651
Nr1d1	*Rn18s*	PBS	156.97	8.700 ± 2.046	3.261 ± 3.105	0.001	0.60
		HDM	195.97	6.205 ± 1.782	1.647 ± 1.425	0.001	0.67
	Rplp0	PBS	215.30	7.907 ± 1.670	4.945 ± 5.236	0.0002	0.6971
		HDM	208.43	6.903 ± 1.597	2.385 ± 2.239	0.0007	0.6973
	Tbp	PBS	210.32	7.900 ± 1.730	4.573 ± 5.139	0.002	0.5875
		HDM	177.37	7.202 ± 1.866	2.092 ± 1.593	8.6E-05	0.79
	Rplp0+Hprt1	PBS	223.32	7.799 ± 1.618	5.060 ± 5.722	0.0008	0.638
		HDM	206.30	6.985 ± 1.605	2.515 ± 2.365	0.0009	0.6893

	Actb	PBS	281.06	7.233 ± 1.183	9.657 ± 16.085	0.02	0.41
		HDM	240.41	6.801 ± 1.289	3.284 ± 3.348	8.5E-05	0.79
Per2	*Rn18s*	PBS	109.55	14.742 ± 2.512	1.554 ± 0.947	0.003	0.5536
		HDM	127.02	12.593 ± 2.510	2.060 ± 1.305	0.001	0.6567
	Rplp0	PBS	131.71	13.626 ± 2.519	2.059 ± 1.262	0.001	0.6125
		HDM	172.67	12.464 ± 2.047	3.22 ± 2.437	5.6E-05	0.8042
	Tbp	PBS	113.23	14.306 ± 2.583	1.942 ± 1.013	0.0001	0.7051
		HDM	175.54	12.898 ± 1.981	3.25 ± 2.592	5E-05	0.8083
	Rplp0+Hprt1	PBS	125.78	13.601 ± 2.582	2.029 ± 1.127	0.0003	0.6753
		HDM	176.20	12.554 ± 2.005	3.462 ± 2.610	9E-06	0.8547
	Actb	PBS	150.77	12.073 ± 2.464	2.762 ± 1.652	9E-06	0.8089
		HDM	178.84	11.620 ± 1.989	3.796 ± 2.730	2E-06	0.8851
Cry2	*Rn18s*	PBS	67.52	16.383 ± 3.055	1.073 ± 0.469	NaN	-0.241
		HDM	45.16	11.480 ± 3.413	1.201 ± 0.507	NaN	-0.1605
	Rplp0	PBS	66.29	13.775 ± 3.153	1.384 ± 0.457	0.006	0.5099
		HDM	88.68	11.879 ± 2.916	1.7 ± 0.675	0.0001	0.762
	Tbp	PBS	51.68	15.062 ± 3.151	1.315 ± 0.367	0.004	0.5432
		HDM	91.56	12.999 ± 2.854	1.682 ± 0.711	1E-06	0.894
	Rplp0+Hprt1	PBS	62.12	13.620 ± 3.207	1.372 ± 0.394	0.002	0.5851
		HDM	95.60	12.098 ± 2.845	1.82 ± 0.769	6.6E-05	0.7991
	Actb	PBS	87.33	10.260 ± 2.945	1.929 ± 0.958	0.001	0.5948
		HDM	65.80	10.167 ± 2.677	2.044 ± 0.855	4E-06	0.8731

Supplementary Table S2. CircWave analysis showing temporal expression of most and least stable housekeeping genes in the lungs

Housekeeping Gene(s)	Treatment Group	Reference Gene(s) (Housekeeping gene used for normalization)	Center of Gravity (COG) ± SD	Data Mean ± SD	*P*-value	r^2
Rn18s	PBS	*Rplp0*	9.409 ± 3.471	1.448 ± 0.556	NaN	-0.2627
		Tbp	12.718 ± 3.646	1.387 ± 0.541	NaN	-0.0632
		Rplp0+Hprt1	8.646 ± 3.440	1.445 ± 0.571	NaN	-0.2946
	HDM	*Rplp0*	10.386 ± 3.071	1.585 ± 0.839	NaN	-0.2303
		Tbp	11.992 ± 3.014	1.567 ± 0.987	NaN	-0.2506
		Rplp0+Hprt1	10.815 ± 3.023	1.666 ± 0.858	NaN	-0.2982
Rplp0	PBS	*Rn18s*	19.722 ± 3.137	0.800 ± 0.322	NaN	-0.2145
		Tbp	18.804 ± 3.408	0.965 ± 0.156	NaN	-0.2111
		Rplp0+Hprt1	18.289 ± 3.641	1.000 ± 0.084	NaN	-0.0475
	HDM	*Rn18s*	1.222 ± 3.190	0.771 ± 0.337	NaN	-0.2597
		Tbp	16.965 ± 3.724	0.996 ± 0.212	0.0368	0.4232
		Rplp0+Hprt1	7.677 ± 3.599	1.062 ± 0.081	NaN	-0.2919
Tbp	PBS	*Rn18s*	19.523 ± 3.251	0.845 ± 0.366	NaN	-0.1064
		Rplp0	13.991 ± 3.704	1.061 ± 0.183	NaN	-0.1828
		Rplp0+Hprt1	17.019 ± 3.476	1.031 ± 0.114	NaN	-0.3506
	HDM	*Rn18s*	2.976 ± 3.064	0.786 ± 0.310	NaN	-0.3334
		Rplp0	5.922 ± 3.225	1.045 ± 0.210	0.0262	0.4551
		Rplp0+Hprt1	6.544 ± 3.267	1.105 ± 0.212	NaN	-0.3884
Actb	PBS	*Rn18s*	20.602 ± 2.707	0.673 ± 0.351	0.0094	0.4866
		Rplp0	19.683 ± 3.071	0.816 ± 0.255	0.0154	0.4491
		Tbp	19.740 ± 2.864	0.795 ± 0.302	0.0025	0.5763
		Rplp0+Hprt1	19.862 ± 3.087	0.805 ± 0.230	0.0054	0.5247
	HDM	*Rn18s*	23.161 ± 2.939	0.664 ± 0.330	0.0095	0.539
		Rplp0	20.971 ± 3.542	0.876 ± 0.232	0.0097	0.5376
		Tbp	19.334 ± 3.318	0.875 ± 0.282	1.4E-05	0.8442
		Rplp0+Hprt1	19.411 ± 3.546	0.928 ± 0.248	0.0045	0.5933
