# Supplementary material for: Evaluation of stable reference genes for qPCR normalization in circadian studies related to lung inflammation and injury in mouse model
Source: Sci Rep. 2022 Feb 2;12:1764. doi: 10.1038/s41598-022-05836-1 (PMC8810972; doi:10.1038/s41598-022-05836-1)
Supplement: Supplementary file 2 — Supplementary Figures. [file 41598_2022_5836_MOESM2_ESM.pdf]

Figure S1

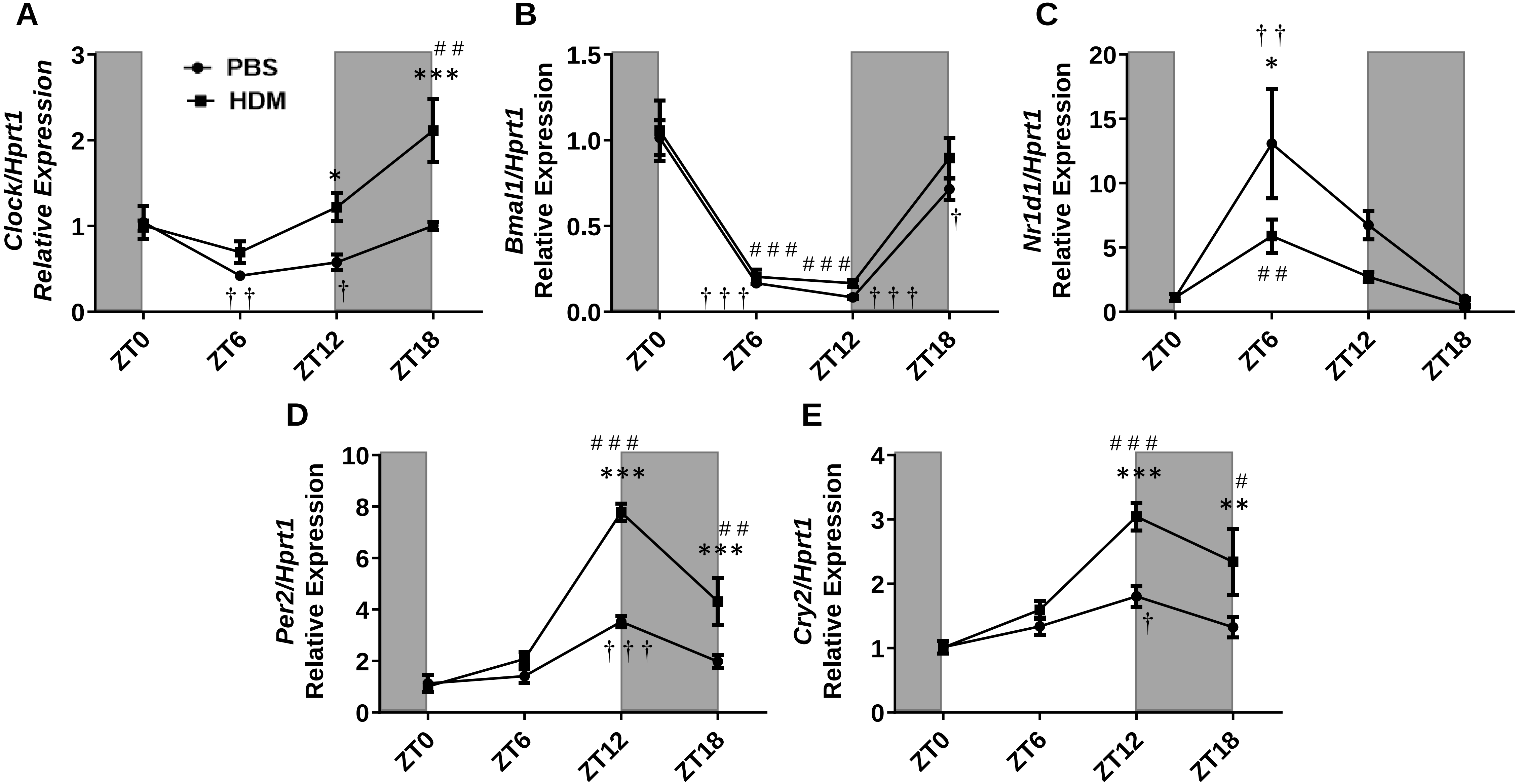

Figure S1. Relative expression of core clock genes in HDM and PBS exposed mouse lungs and data normalized with *Hprt1* reference gene. (A) *Clock*, (B) *Bmal1*, (C) *Nr1d1*, (D) *Per2* and (E) *Cry2* relative expression. Symbols † and # represent the statistical significance in temporal expression of circadian clock genes in the PBS and HDM group respectively at the marked time point when compared to the baseline (ZT0). White and gray areas in the graph represents the light and dark cycle, respectively. Data are shown as mean ± SEM (n= 4/group; * $P < 0.05$, ** $P < 0.01$ and *** $P < 0.001$ compared to respective control).

Figure S2

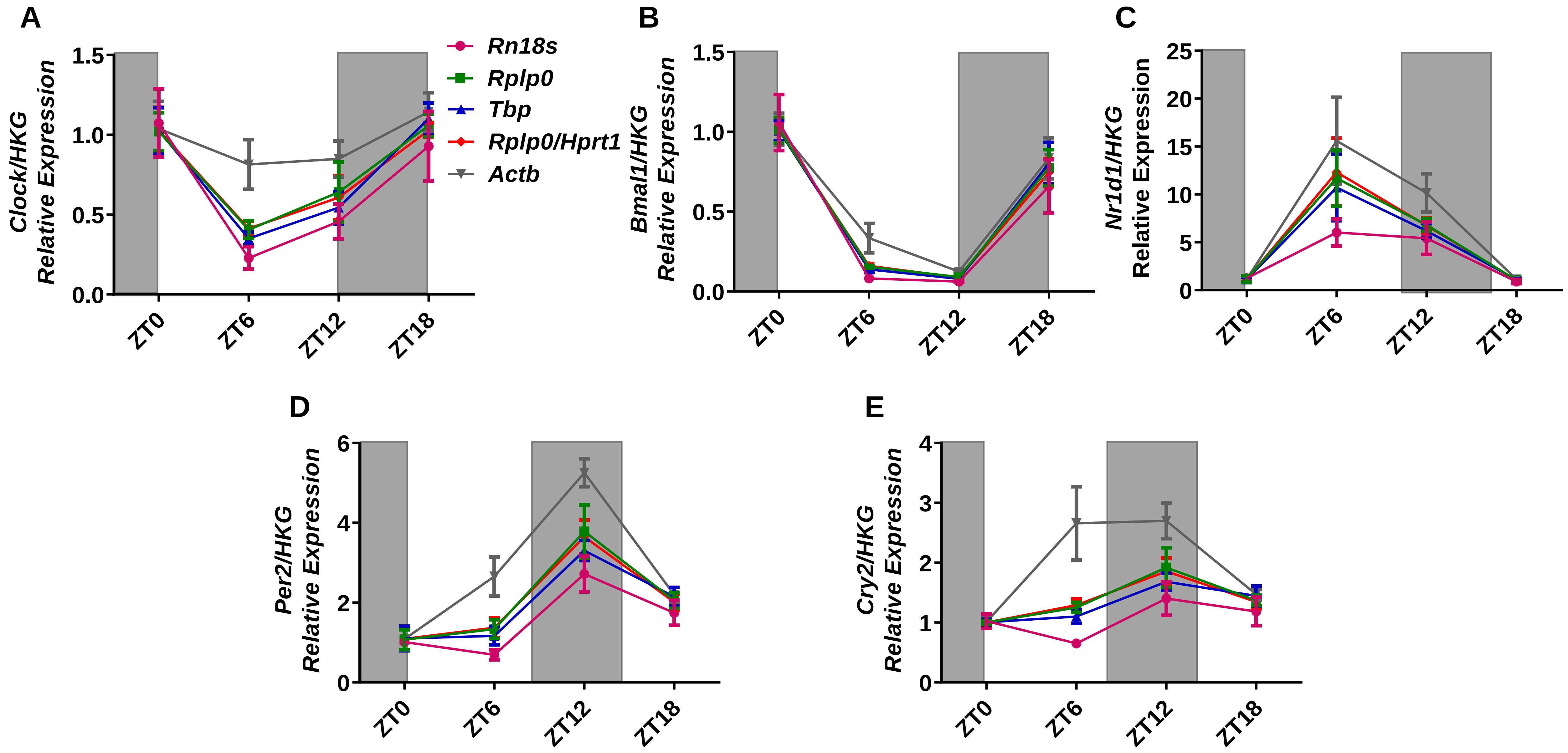

Figure S2. Summary of the relative expression of the circadian clock genes evaluated in the PBS (control) group using different reference genes. (A) *Clock*, (B) *Bmal1*, (C) *Nr1d1*, (D) *Per2* and (E) *Cry2* relative expression in PBS group. White and gray areas in the graph represents the light and dark cycle, respectively. Data are shown as mean ± SEM (n= 4/group).

Figure S3

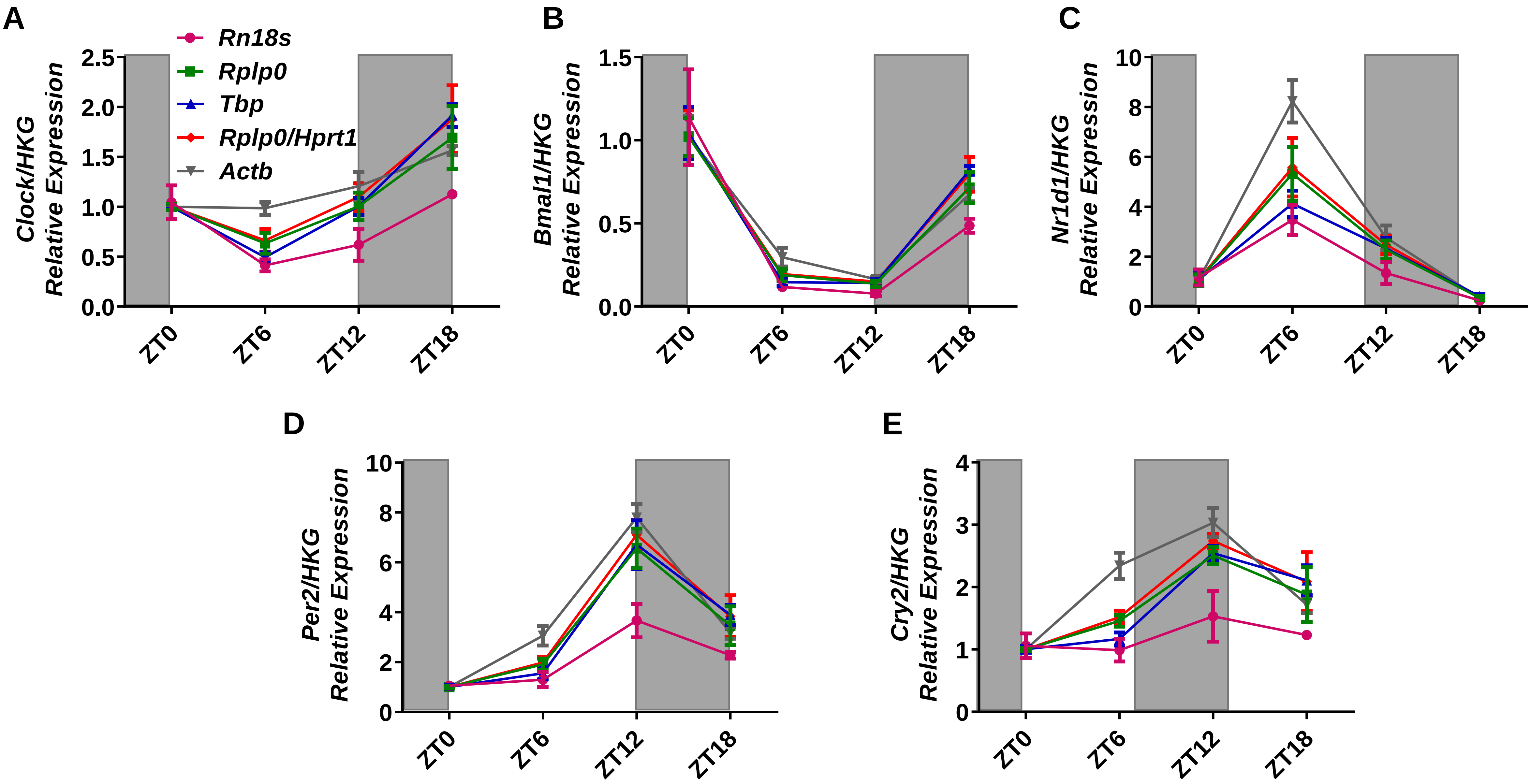

Figure S3. Summary of the relative expression of the circadian genes evaluated in the acute HDM exposed group using different normalizer genes. (A) *Clock*, (B) *Bmal1*, (C) *Nr1d1*, (D) *Per2* and (E) *Cry2* relative expression in HDM exposed group. White and gray areas in the graph represents the light and dark cycle, respectively. Data are shown as mean ± SEM (n= 4/group).

Figure S4

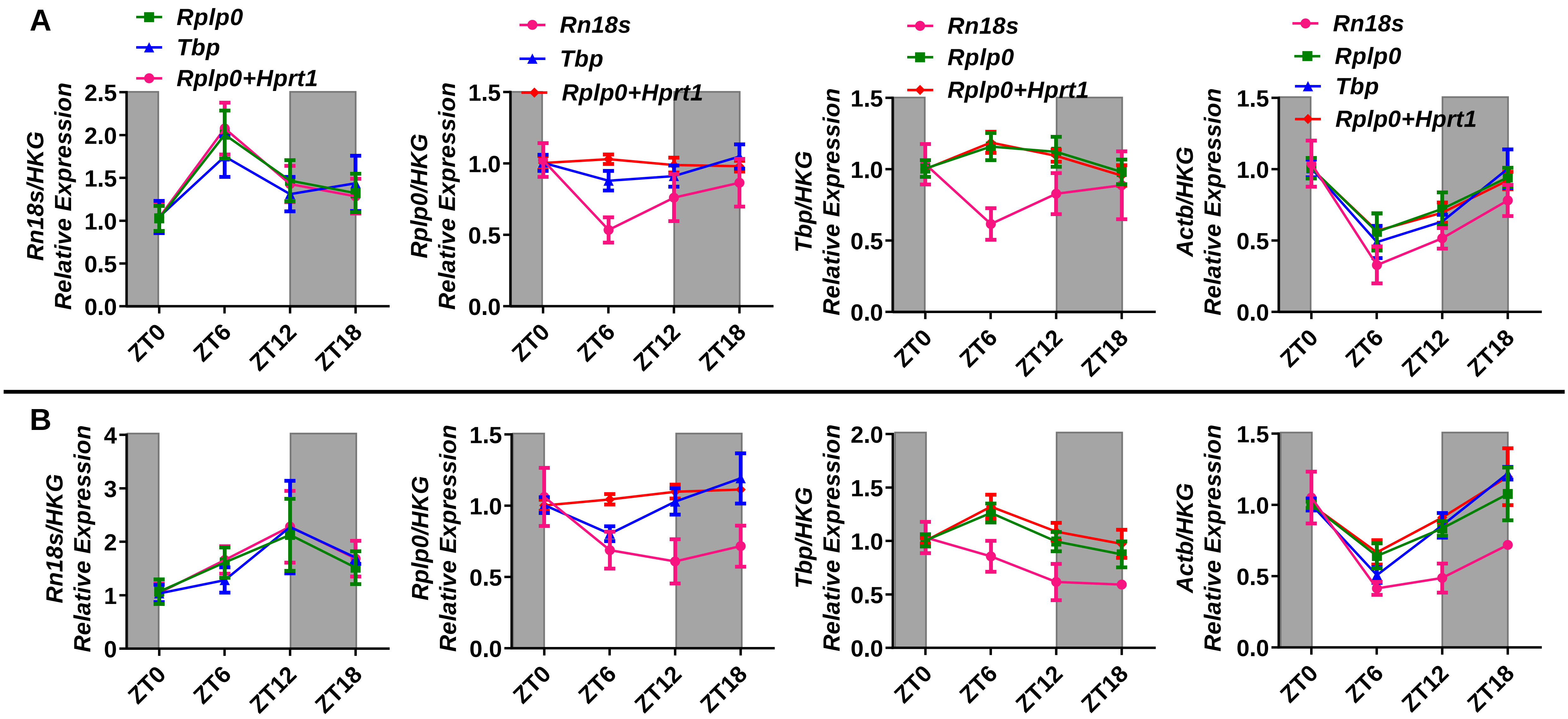

Figure S4. Summary of the temporal expression of most and least stable housekeeping genes evaluated in PBS and HDM exposed groups using different housekeeping genes as normalizers. The relative expression of the *Rn18s, Rplp0, Tbp* (most stable HKG) and *Actb* (least stable HKG) in (A) PBS and (B) HDM groups were plotted separately. White and gray areas in the graph represents the light and dark cycle, respectively. Data are shown as mean ± SEM (n= 4/group).
